# Controlling superstructural ordering in the clathrate-I Ba_8_M_16_P_30_ (M = Cu, Zn) through the formation of metal–metal bonds†
†Electronic supplementary information (ESI) available: Experimental details, tables of crystal structure refinements and structural parameters and associated CIFs, synchrotron X-ray and neutron powder diffraction Rietveld refinement plots, additional TEM and elemental analysis figures, LMTO DOS and band structure diagrams. See DOI: 10.1039/c7sc00354d



**DOI:** 10.1039/c7sc00354d

**Published:** 2017-02-20

**Authors:** J. Dolyniuk, P. S. Whitfield, K. Lee, O. I. Lebedev, K. Kovnir

**Affiliations:** a Department of Chemistry , University of California , Davis , One Shields Avenue , Davis , CA 95616 , USA . Email: kkovnir@ucdavis.edu; b Chemical and Engineering Materials Division , Oak Ridge National Laboratory , Oak Ridge , Tennessee 37830 , USA; c Laboratoire CRISMAT , ENSICAEN , CNRS UMR 6508 , 6 Boulevard du Mareéchal Juin , F-14050 Caen , France

## Abstract

Order–disorder–order phase transitions in the clathrate-I Ba_8_Cu_16_P_30_ were induced and controlled by aliovalent substitutions of Zn into the framework. Unaltered Ba_8_Cu_16_P_30_ crystallizes in an ordered orthorhombic (*Pbcn*) clathrate-I superstructure that maintains complete segregation of metal and phosphorus atoms over 23 different crystallographic positions in the clathrate framework. The driving force for the formation of this *Pbcn* superstructure is the avoidance of Cu–Cu bonds. This superstructure is preserved upon aliovalent substitution of Zn for Cu in Ba_8_Cu_16–*x*_Zn_*x*_P_30_ with 0 < *x* < 1.6 (10% Zn/M_total_), but vanishes at greater substitution concentrations. Higher Zn concentrations (up to 35% Zn/M_total_) resulted in the additional substitution of Zn for P in Ba_8_M_16+*y*_P_30–*y*_ (M = Cu, Zn) with 0 ≤ *y* ≤ 1. This causes the formation of Cu–Zn bonds in the framework, leading to a collapse of the orthorhombic superstructure into the more common cubic subcell of clathrate-I (*Pm*3*n*). In the resulting cubic phases, each clathrate framework position is jointly occupied by three different elements: Cu, Zn, and P. Detailed structural characterization of the Ba–Cu–Zn–P clathrates-I *via* single crystal X-ray diffraction, joint synchrotron X-ray and neutron powder diffractions, pair distribution function analysis, electron diffraction and high-resolution electron microscopy, along with elemental analysis, indicates that local ordering is present in the cubic clathrate framework, suggesting the evolution of Cu–Zn bonds. For the compounds with the highest Zn content, a disorder–order transformation is detected due to the formation of another superstructure with trigonal symmetry and Cu–Zn bonds in the clathrate-I framework. It is shown that small changes in the composition, synthesis, and crystal structure have significant impacts on the structural and transport properties of Zn-substituted Ba_8_Cu_16_P_30_.

## Abstract 2

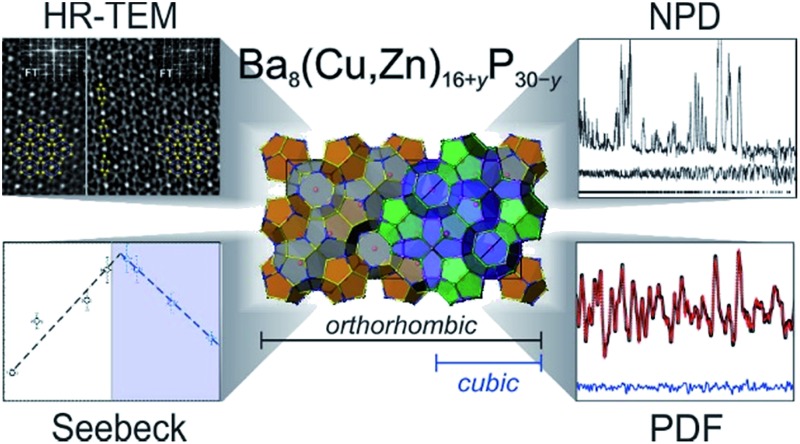
Formation and avoidance of M–M bonds in the clathrate framework result in extreme structural complexity.

## Introduction

In materials science, substitutional doping is often used to adjust samples’ charge carrier concentration and transport properties. The exact locations and bonding of aliovalent dopants are often assumed and not further characterized due to the inherent difficulties substitutions can present, such as low concentrations of dopants or similar X-ray scattering factors of neighboring elements. In this work, we show the importance of proper, albeit challenging, structural characterization to obtain an accurate description of a doped thermoelectric material. Thermoelectric cells hold promise for energy harvesting applications; they provide the potential to convert wasted heat into usable electrical energy. Such cells have been implemented in space technologies for decades, and they can now be seen in portable coolers, wine chillers, and other niche markets. So far, the efficiencies of known TE materials have not been sufficient for widespread production and use.^1^ Thus, the search continues for newer, better thermoelectrics. Thermoelectric efficiency is characterized by the dimensionless, temperature-dependent figure of merit, calculated by the formula *ZT* = *S* ^2^ *T*/*ρκ*. In this equation, *T* is the absolute temperature, *S* is the Seebeck thermopower, *ρ* is the electrical resistivity, and *κ* is the thermal conductivity. The greatest obstacle hindering the development of high *ZT* materials is the strong coupling of *S*, *ρ*, and *κ*.^1^

The phonon-glass electron-crystal (PGEC) idea suggests the decoupling of heat and charge transport using ordered frameworks and guest atoms with large displacement parameters: “rattlers”. Inorganic clathrates are PGEC materials. Their ordered covalent frameworks with large polyhedral cages can host guest cations that donate their valence electrons to the framework, providing an electron-balanced, semiconducting Zintl system, as required for thermoelectrics. This tetrahedral coordination is very suitable for tetrels, which are the group 14 elements (Si, Ge, and Sn). More than 200 tetrel clathrates have been discovered.^2,3^ Improvements in their thermoelectric properties have been realized by the partial replacement of tetrel framework atoms with transition metals.^2–6^ This prompted our search for new clathrates with high concentrations of transition metals in their frameworks.

Ba_8_Cu_16_P_30_ is one such clathrate, originally discovered in the 1990s by Mewis *et al.* ^7^ It crystallizes in an orthorhombic *Pbcn* superstructure, where Ba atoms are encapsulated in Cu/P cages with segregated Cu and P sites. In 2003, the thermoelectric properties of Ba_8_Cu_16_P_30_ were characterized and a metallic dependence of the electrical resistivity was observed, indicating the necessity of altering the electronic properties to generate a semiconductor.^8^ We scrutinized a supposedly simple Zn substitution into the Cu sublattice, which proved to be extremely complex from structural and bonding points of view. In the present work, we report a comprehensive characterization of the crystal and electronic structures of the Zn-substituted Ba_8_Cu_16_P_30_ clathrate over its full substitution range, 0–35%, of the total metal content, Zn/(Cu + Zn).

## Experimental

### Synthesis

All manipulations of the starting materials were performed inside an argon-filled glove box (*p*(O_2_) < 1 ppm). The starting materials, metallic barium (Sigma-Aldrich, 99.9%), copper powder (Alfa Aesar, 99.99%), zinc shavings (Alfa Aesar, 99.8%), and red phosphorus (Alfa Aesar, 99%), were used as received.

All samples of Ba_8_Cu_16–*x*_Zn_*x*_P_30_ were obtained from solid state reactions of the elements. Single phase samples of Ba_8_Cu_16–*x*_Zn_*x*_P_30_ (0 < *x* < 3) were obtained from stoichiometric mixtures of elemental Ba, Cu, Zn, and P for *x* = 0, 0.5, 1, 1.5, 1.75, 2, 2.25, 2.5, and 3 with a total mass of 1 g of starting materials. Samples of *x* ≈ 4 (24.2% Zn/M_total_) and *x* ≈ 5 (29.4% Zn/M_total_, M_total_ = Zn + Cu) were synthesized using slightly P-deficient stoichiometries of Ba_8_Cu_12.5_Zn_4_P_29.5_ and Ba_8_Cu_12_Zn_5_P_29_, respectively. A sample of maximum Zn content (*x* = 5.6, 35% Zn/M_total_) was synthesized using the Ba_8_Cu_10.4_Zn_5.6_P_30_ nominal composition. Samples were either placed in glassy-carbon crucibles inside silica ampoules or placed directly into carbonized silica ampoules. In both cases the ampoules were evacuated and flame-sealed. The ampoules were heated to 1173 K over 17 h, annealed at this temperature for 72 h, and then cooled to room temperature. The products were ground in the glovebox and re-annealed at 1173 K for 140 h, cooled down, reground in the glovebox and re-annealed under the same conditions for another 140 h.

The melting temperatures of all synthesized clathrates were determined using differential scanning calorimetry. To ensure homogeneity, all samples were subsequently heated above the melting temperature in two different ways. The samples with *x* < 1.5 (9.4% Zn/M_total_) were heated to 1223 K over 5 hours, held there for 10 hours, and then allowed to cool to room temperature. Samples with intermediate Zn content, 1.5 < *x* < 2.5 (9.5–16% Zn/M_total_), were heated to 1223 K over 5 hours, held there for 10 hours, and air-quenched by removing the ampoules from the furnace. Samples with high Zn content, *x* > 2.5 (>16% Zn/M_total_), were heated to 1173 K over 5 hours, held there for 10 hours, and air-quenched by removing the ampoules from the furnace. This complex quenching procedure was developed as the result of multiple quenching and melting experiments in order to avoid or minimize impurity formation and significant losses of Zn due to vaporization.

### Characterization

The synthesized clathrate samples were characterized using single crystal X-ray diffraction, synchrotron high-resolution and laboratory powder X-ray diffraction (XRD), time-of-flight neutron powder diffraction, X-ray and neutron pair distribution function (PDF) analyses, energy dispersive X-ray spectroscopy (EDXS), electron diffraction, high-angle annular dark field scanning TEM (HAADF-STEM) and annular bright field scanning TEM (ABF-STEM), quantum chemical calculations, and transport properties measurements. Detailed characterization and experimental details are provided in the ESI.†

## Results and discussion

**Fig. 1:**
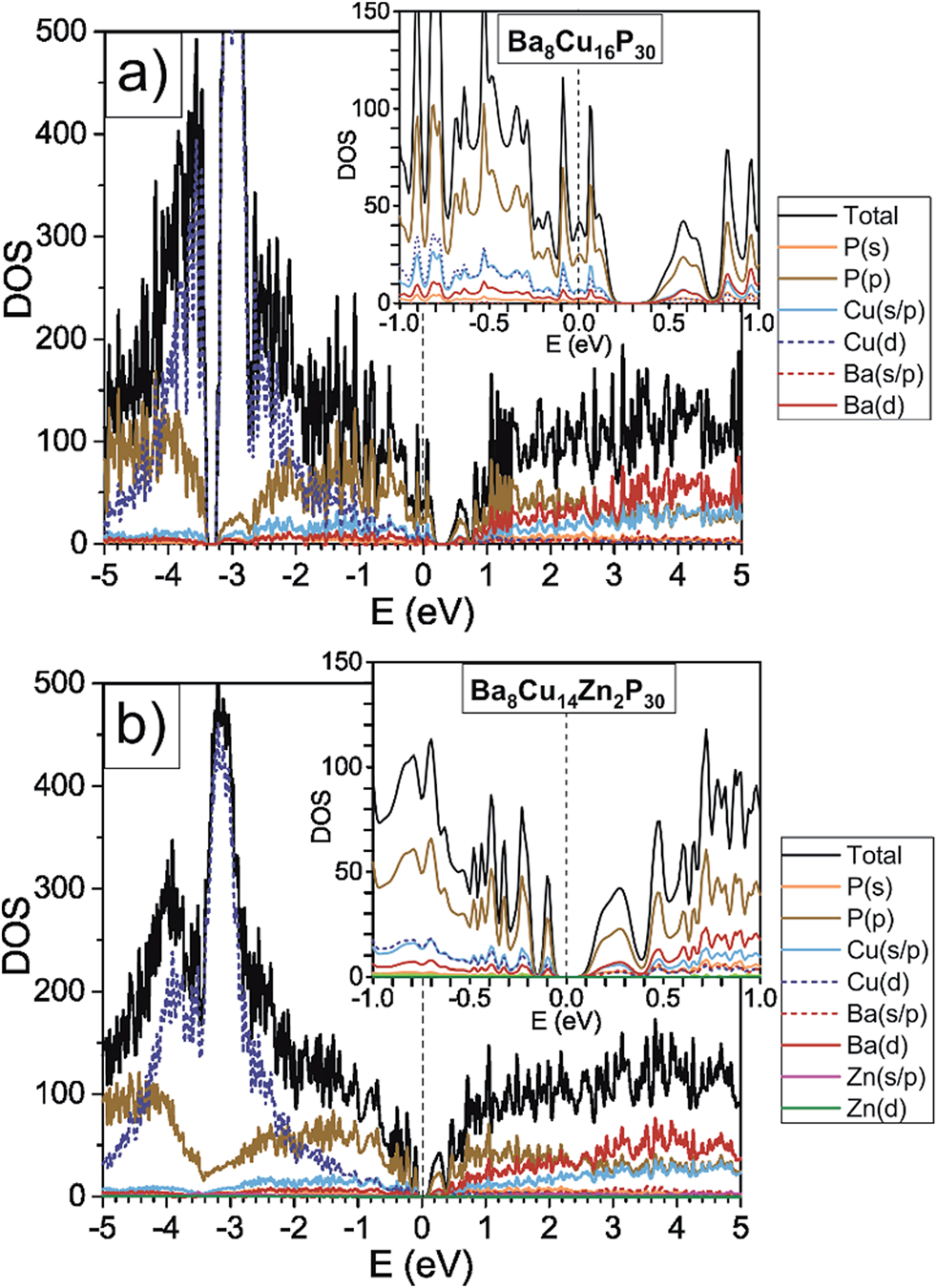
GGA-PBE density of states calculations are shown for orthorhombic Ba_8_Cu_16_P_30_ and one possible model of Ba_8_Cu_14_Zn_2_P_30_, where Zn solely occupies one crystallographic position, and Cu–Zn bonding is avoided. The density of states for Ba_8_Cu_16_P_30_ was reproduced by a second computational method, TB-LMTO-ASA, Fig. S2.†

Ba_8_Cu_16_P_30_ has a complex structure and a large unit cell, which causes low thermal conductivity. This compound is also metallic, and therefore not ideal for thermoelectric applications.^8^ In this material, Cu and P are segregated over different crystallographic sites, leading to an orthorhombic supercell of the common cubic clathrate-I subcell.^7,9^ According to the Zintl concept^2,10^ in Ba_8_Cu_16_P_30_, each Ba atom donates two electrons to the framework to become Ba^2+^, and each framework atom requires four electrons for the formation of four covalent bonds, *i.e.* 46 × 4 = 184 electrons per formula unit. Cu and P are expected to provide one and five valence electrons, respectively. The total number of electrons per formula unit is 8 × 2 (Ba) + 16 × 1 (Cu) + 30 × 5 (P) = 182 electrons, which is two electrons fewer than what is required by the four-coordinate Zintl counting scheme.^2^ This electron count is supported by density of states calculations, which show that Ba_8_Cu_16_P_30_ is a metal with a Fermi level located in the valence band (Fig. 1, top). Positive Seebeck coefficients for Ba_8_Cu_16_P_30_ over the whole measured temperature range confirm that holes are the main charge carriers.^8^

**Fig. 2:**
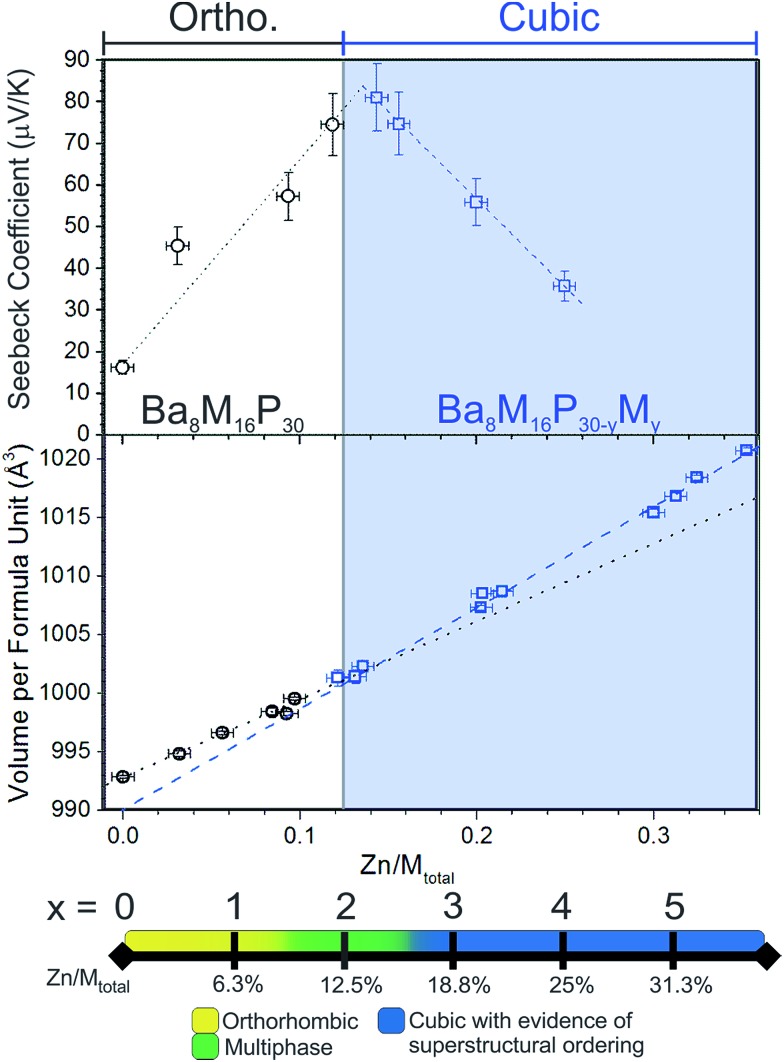
(Top) Room temperature Seebeck coefficients for selected samples of Ba–Cu–Zn–P clathrate-I. The maximum Seebeck coefficient is observed around the expected semiconducting stoichiometry, Ba_8_Cu_14_Zn_2_P_30_, with 12.5% Zn/M_total_. White and blue backgrounds approximately correspond to phase regions with orthorhombic and cubic structures of the clathrate phases, respectively. (Middle) A plot of Zn content *versus* unit cell volume per formula unit for selected single crystals. Volumes of orthorhombic and cubic crystals are represented by black circles and blue squares, respectively. Dashed and dotted lines are linear fits of the volume *vs.* Zn content dependencies for cubic and orthorhombic structures, respectively. Note different slopes of the dashed and dotted lines. (Bottom) A visual summary of the results of powder X-ray diffraction techniques (PXRD) is presented as a scale bar of increasing Zn content.

Based on Zintl counting, the replacement of two Cu atoms with Zn atoms (2 extra electrons, 12.5% Zn/M_total_) in the Ba_8_Cu_16_P_30_ structure is expected to result in a charge-balanced compound with semiconducting properties. This prediction is supported by density of states calculations, which suggest that an additional 2 electrons per formula unit will move the Fermi level to the top of the valence band (Fig. 1, bottom). The maximum values of experimentally determined Seebeck coefficients over the range of measured compositions are reached for compositions with close to 2 Zn atoms (12.5% Zn/M_total_) (Fig. 2, top).

According to the Zintl count and the calculated density of states, an increase in the Zn content of over two atoms per formula unit should result in the formation of an n-type material due to the relocation of the Fermi level into the conduction band. However, this is not the case. The Seebeck coefficients remain positive for all samples, irrespective of the Zn content.

By synergistically applying a number of characterization techniques, we determined that for samples with high Zn content, over 12.5% Zn/M_total_, instead of forming the Ba_8_Cu_16–*x*_Zn_*x*_P_30_ phase, a more complex phase, Ba_8_M_16+*y*_P_30–*y*_ (M = Cu, Zn) (0 ≤ *y* ≤ 1), forms in which the P content is reduced below 30 atoms per formula unit. Considering the Zintl description, every replacement of P with Zn will lead to 3 fewer electrons. This suggests that the composition Ba_8_Cu_11_Zn_6_P_29_ should be electron balanced, 8 × 2 (Ba) + 11 × 1 (Cu) + 6 × 2 (Zn) + 29 × 5 (P) = 184 electrons, which is exactly 4 electrons per framework atom. We hypothesize that the P-deficient, M-rich phases ultimately result from the formation of Cu–Zn bonds in the structure. Since the avoidance of Cu–Cu interactions led to the realization of the orthorhombic supercell in Ba_8_Cu_16_P_30_,^9^ Cu–Zn bonds are more likely than the formation of Cu–Cu bonds.

Consequently, further addition of Zn atoms over 12.5% Zn/M_total_ not only led to P-deficient phases, but also triggered a structural conversion of the primary orthorhombic √2 × 1 × 2√2 *Pbcn* cell (*a* ≈ 14 Å, *b* ≈ 10 Å, *c* ≈ 28 Å, *V* ≈ 4000 Å^3^) into a cubic *Pm*3*n* subcell (*a* ≈ 10 Å, *V* ≈ 1000 Å^3^). In this higher symmetry subcell, the previously segregated metal and phosphorus sites merge into mixed occupancy sites. Detailed structural investigation of the samples with high Zn content indicates that these compounds are not completely disordered, but are instead locally ordered, merely lacking complete long-range ordering.

### Electronic structure and Seebeck coefficients

Density of states calculations for Ba_8_Cu_16_P_30_ and Ba_8_Cu_14_Zn_2_P_30_ show that the states near the Fermi levels and bandgaps are predominantly composed of P 3p-, Cu 4s-, 4p-, and 3d-, and Ba 5d-orbitals. In Ba_8_Cu_14_Zn_2_P_30_, Zn orbitals do not contribute much to the region near the Fermi level, thus Zn mainly increases the number of electrons in the system. The calculated bandgap of Ba_8_Cu_14_Zn_2_P_30_ is 0.1 eV, which is smaller than the separation between the conduction and valence bands calculated for Ba_8_Cu_16_P_30_, 0.2 eV.

Powdered single-phase samples of Zn-substituted Ba_8_Cu_16_P_30_ were compacted using spark plasma sintering. The resulting geometrical densities of the pellets were greater than 90% of their theoretical X-ray densities. Seebeck coefficients were measured for selected single-phase samples ranging from Ba_8_Cu_16_P_30_ to Ba_8_M_16+*y*_P_30–*y*_ with 25% Zn/M_total_ (Fig. 2, top). In addition to these samples, selected samples of mixed orthorhombic and cubic structures (1.5 ≤ *x* < 3) were also measured. The Seebeck coefficients remained positive in the 5–400 K temperature range for all samples. The maximum Seebeck coefficient is observed around the expected semiconducting stoichiometry, Ba_8_Cu_14_Zn_2_P_30_, with a room temperature value of nearly 80 μV K^–1^.

### Single crystal X-ray diffraction

The detailed structural data are shown in Tables S1–S4.† The unit cell volume increases upon replacement of Cu with Zn (Fig. 2, middle). An approximate 3% increase in the total volume per formula unit is observed between Ba_8_Cu_16_P_30_ and the sample with the highest Zn content, Zn/M_total_ = 35%. This volume increase is not surprising, as the replacement of Zn with Cu in solids is known to lead to an increase in unit cell volume as in the case of the isostructural phases CaM_2_Ge_2_ (M = Cu, Zn) where the Zn analogue is 10% larger than the Cu analogue.^11^

In Fig. 2 (middle), the linear fits are plotted for Zn content *versus* volume per formula unit for cubic and orthorhombic crystals. The slopes of these lines are different. The rate of increase in volume with Zn content for orthorhombic systems is lower than that of the cubic systems. This is expected if only the Cu framework atoms are replaced with the larger Zn atoms in the orthorhombic systems, but both the Cu and the smaller P framework atoms are replaced by the larger Zn atoms above *x* ≈ 2 (≈12.5% Zn/M_total_) in the cubic systems.

### High-resolution synchrotron X-ray powder diffraction

**Fig. 3:**
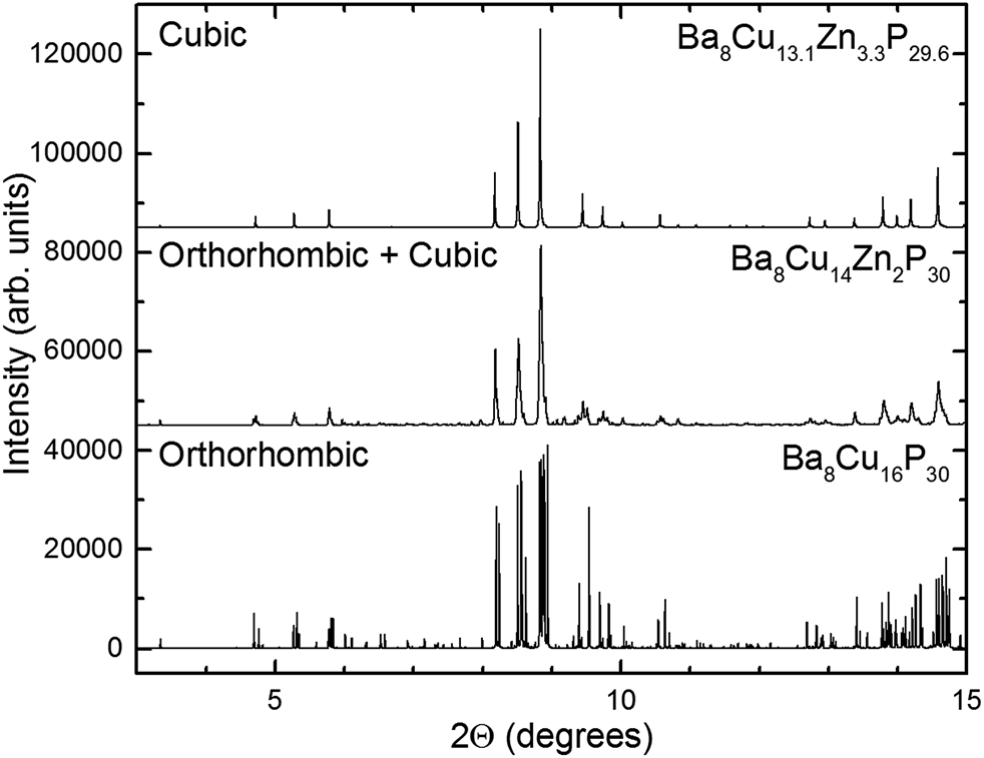
Synchrotron X-ray powder diffraction patterns for the cubic (top), orthorhombic (bottom), and mixed orthorhombic and cubic (middle) samples. Data were collected at 100 K and an average wavelength of 0.41 Å.

High-resolution synchrotron powder X-ray diffraction data were acquired and analyzed over the range of stoichiometries. The results of those measurements were the following: samples of nominal 0 ≤ *x* < 1.5 were mainly orthorhombic (Fig. 3, bottom), at nominal 1.5 ≤ *x* ≤ 2.125, the main orthorhombic peaks began to merge into single peaks (Fig. 3, middle), and at nominal *x* = 2.5, the peaks fused into broad cubic peaks with shoulders. These shoulders are also present in the nominal 3 Zn sample (actual composition Ba_8_Cu_13.1_Zn_3.3_P_29.6_) but with smaller intensities (Fig. 3, top). Nominal 4 and 5 Zn samples show sharp cubic peaks with no shoulders. An increase in the unit cell volume with the addition of Zn is emphasized by a significant shift of the diffraction peaks to lower 2*θ* angles.

### Crystal structure

**Fig. 4:**
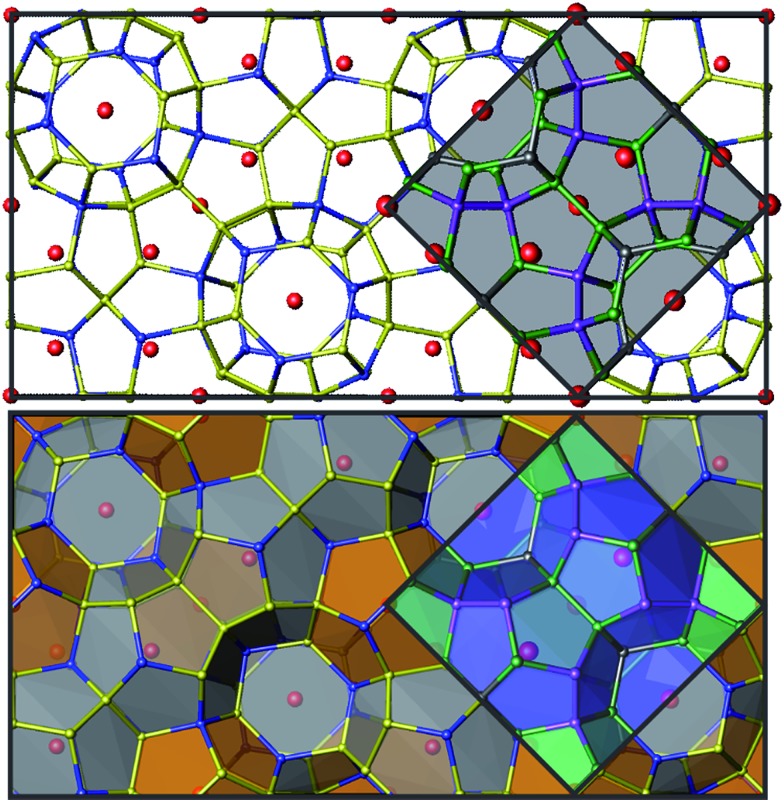
Top: unit cells are shown for the orthorhombic cell (black) and the cubic subcell (gray shading). Bottom: polyhedral representations of the orthorhombic and cubic unit cells are shown with the two cage types. Barium atoms are shown in red, M atoms (M = Cu, Zn) are blue, and P atoms are yellow for the orthorhombic cell. In the cubic cell, 6c sites are shown in dark gray, 16i sites are purple, and 24k sites are green. See Fig. S1† for additional details.

Superstructures and partial ordering are well-known in clathrates-I, which display a variety of unit cell symmetries.^2,12–17^ All samples of Zn-substituted Ba_8_Cu_16_P_30_ crystallize in a clathrate-I type structure, composed of Ba-stuffed small dodecahedra and large tetrakaidecahedra (Fig. 4). At low Zn content in Ba_8_M_16_P_30_ (M = Cu, Zn), orthorhombic superstructural ordering leads to the segregation of M and P atoms over different crystallographic sites. This generates five unique Ba positions, eight Cu positions, and fifteen P positions (Fig. S1†). Upon further Zn substitution, the cubic Ba_8_M_16+*y*_P_30–*y*_ (M = Cu, Zn) phases are formed, in which all framework sites are jointly occupied by M and P atoms. This unit cell is on the order of one quarter of the size of the orthorhombic superstructure (Fig. 4: gray diamond). Following this conversion of the orthorhombic *Pbcn* cell to the cubic *Pm*3*n* cell, the 23 Cu and P sites are converted into three mixed occupancy M/P sites. In the cubic cell, the 6c site evolves from one Cu and two P sites; the 16i site evolves from two Cu and six P sites; and the 24k site evolves from five Cu and seven P sites (Fig. S1†).^9^

For the two samples with 31% Zn/M_total_ (nominal Zn = 5), a rhombohedral cell, *R*3*c*, was observed. The primitive part of the rhombohedral unit cell has an identical volume and number of atoms to the cubic cell. Reduction of the symmetry from cubic to rhombohedral resulted in the formation of two unique Ba crystallographic sites, four mixed M/P sites, and one fully occupied P site. The latter one forms a short P–P bond to itself (2.21 Å), which is too short for a M–P bond.^18–22^ This is not the first observation of a rhombohedral distortion in a clathrate-I system. In 2004, Cros *et al.* reported the existence of a Si–Te clathrate-I with *R*3*c* symmetry. In their case, three cubic *Pm*3*n* positions split into six Si and two Te positions upon substitution of Te into the Si clathrate framework.^23^

Since all of the Ba_8_M_16_P_30_ and Ba_8_M_16+*y*_P_30–*y*_ (M = Cu, Zn) compounds retain a common clathrate-I structure, the Ba atoms in the small pentagonal dodecahedra coordinate to 20 framework atoms, and those in the larger tetrakaidecahedra coordinate to 24 framework atoms. A significant increase in the shortest Ba-framework distance is seen for the higher Zn content systems (Table S4†). The shortest Ba-framework (Ba–P) distances for the orthorhombic clathrates are 3.16–3.17 Å. This distance increases substantially to 3.21–3.24 Å in the cubic/rhombohedral systems, and at the highest Zn content, Ba vacancies are formed. Similar vacancy formation in a guest site was seen in the cationic Si–P–Te clathrates where the guest Te atoms preferentially vacate cages with the highest concentration of P.^24,25^

The shortest Ba–Ba distances also increase from a range of 4.83–4.84 Å in the orthorhombic systems to 5.00–5.03 Å in the cubic systems. These distances are similar to the ones found in other Ba-containing Zintl phases.^26,27^ Selected bond distances and angles are shown in Table S4.† The orthorhombic-to-cubic conversion also affects P–P bonding. An extra-long P–P distance that is present in the orthorhombic structures remains almost unchanged with Zn substitution: in Ba_8_Cu_16_P_30_ and Ba_8_Cu_14.4_Zn_1.6_P_30_ the distances are 2.477(2) Å and 2.470(2) Å, respectively. For the cubic phase with *x* close to 2, there is a large drop in the longest framework–framework distance down to 2.401(3) Å between two atoms in the 24k site.

**Fig. 5:**
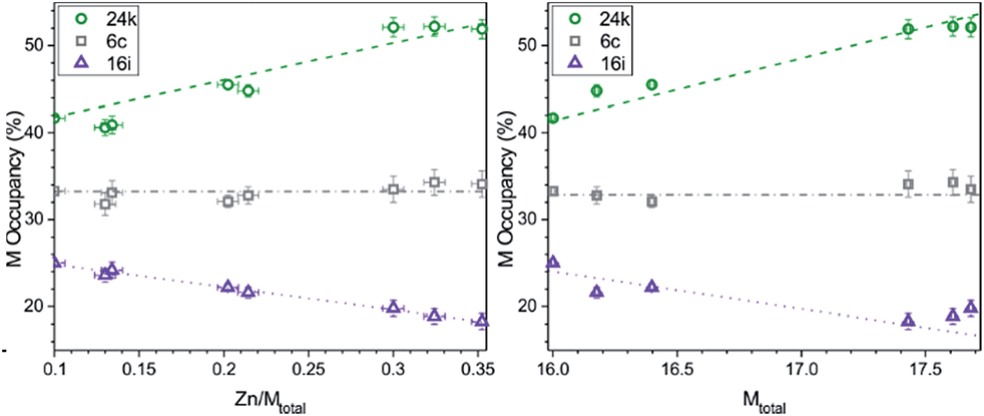
The M occupancy (M = Cu + Zn) for each of the cubic framework sites: 24k (green circle), 6c (gray square), and 16i (purple triangle) per Zn/M_total_ (left) and per total M content (right). Lines are added to guide the eyes.

With the increase of Zn content, the amount of metal does not increase equally in all cubic framework sites. As shown in Fig. 5, at the expected semiconducting composition of ∼2 Zn (12.5% Zn/M_total_), the metal (M = Cu, Zn) atoms show a preference for the 24k site (black circles), where nearly half of the sites are occupied by M atoms. In contrast, smaller P atoms preferentially fill up the 16i site (red triangles) which has the lowest M occupancy. A steady decrease in the M occupancy of the 16i sites can be seen along with an equally steady increase in the M occupancy of the 24k sites with increasing Zn content. The M occupancies of the 6c sites remain nearly constant across all cubic samples. Overall, the longest average bond distances are of the 24k–24k type, and the smallest average bond distances are of 16i–16i type, in accordance with the lowest and highest M occupancies in the 16i and 24k sites, respectively.

This preferential distribution of the M and P atoms suggests the possibility of Cu/Zn ordering. For example, in the crystal structure of the cubic clathrate Ba_8_Cu_4_Si_42_, Cu was found to substitute for Si only in one framework site.^28^ Neutron diffraction was employed to determine any site preferences for the metal atoms in the Ba_8_M_16_P_30_ system.

### Neutron powder diffraction and Rietveld refinements

Time-of-flight (TOF) neutron powder diffraction data was collected for an unsubstituted orthorhombic Ba_8_Cu_16_P_30_ sample, and for Zn-substituted samples of orthorhombic and cubic symmetries with nominal compositions Ba_8_Cu_15_Zn_1_P_30_ and Ba_8_Cu_13_Zn_3_P_30_. Unlike in X-ray diffraction, Cu and Zn are distinguishable *via* neutron diffraction since they have different neutron scattering lengths: 7.7 fm (Cu) and 5.7 fm (Zn), respectively. However, Zn and P (5.1 fm) have similar neutron scattering lengths. Thus, we performed joint neutron and synchrotron X-ray Rietveld refinements (Fig. S3–S5 and Tables S4–S8†).

For the orthorhombic compound of nominal Zn = 1, free refinement of Zn in every Cu atomic position resulted in the composition Ba_8_Cu_15.1_Zn_0.9(2)_P_30_. Instead of having Zn in every site, Zn showed a strong preference for only few specific sites (Table S6†). Zn is consistently found in three sites, Cu1, Cu4, and Cu5. The highest Zn concentration is found in the Cu1 and Cu4 sites with the shortest Ba–M distances, and the shortest (Cu1) and third shortest (Cu4) M–P bonds. Thus, there is more at play than just atomic sizes. Ba–M bonding interactions have been observed in clathrate-I systems, *i.e.* Ba_8_Au_5.3_Ge_40.7_,^4^ and without further calculations cannot be ruled out as a possibility in Ba–Cu–Zn–P clathrates. Note that the orthorhombic Cu1 and Cu4 sites are derived from the 16i sites in a cubic unit cell.

A joint unconstrained refinement of the cubic sample was unstable, so the refinement was performed using the constrained composition of Ba_8_Cu_13.1_Zn_3.3_P_29.6_, determined from elemental analysis and a single crystal X-ray refinement. This refinement indicated that Zn atoms substitute into all three framework positions of the cubic Ba_8_Cu_13.1_Zn_3.3_P_29.6_ structure. The Cu/Zn ratios were determined to be 2 : 1, 2 : 1, and 6 : 1 for the 6c, 16i, and 24k cubic sites, respectively. Zn prefers to occupy the 6c and 16i sites, representing almost a third of the metal content in each site, while Cu occupancy dominates in the 24k site where the Zn content is minimal.

### Superstructural ordering in compositions with high Zn content

**Fig. 6:**
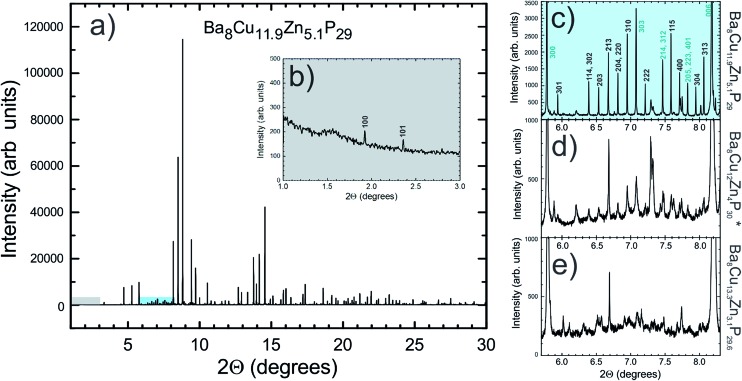
Experimental synchrotron X-ray powder diffraction data for a sample with Zn = 5.1 is shown in (a). The inset (b) shows the fragment (highlighted in grey) of the main pattern with two indexed low angle trigonal peaks clearly visible (100 and 101, primitive trigonal, *a* = 14.2 Å, *c* = 17.4 Å), which should be systematically absent for the rhombohedral and cubic cells. The panel (c) shows the fragment (highlighted in cyan) of the main pattern with multiple diffraction peaks (*I* < 3% of *I*_max_), which can only be indexed in a primitive trigonal cell. The *hkl* indices for the diffraction peaks corresponding to the rhombohedral cell are highlighted in blue. Finally, (d) and (e) show similar regions in the diffraction patterns of samples with nominal Zn content of 4 and 3, respectively. Data were collected at 100 K with an average *λ* = 0.41 Å. *The composition Ba_8_Cu_12_Zn_4_P_30_ is nominal.

Though single crystals of the Ba_8_M_16+*y*_P_30–*y*_ samples were refined in cubic *Pm*3*n* cells, synchrotron powder X-ray diffraction data showed a large set of unindexed low intensity peaks (Fig. 6). These peaks did not match the theoretically calculated *Pm*3*n* powder pattern, but had similar *Q*-spacings and similar full-widths at half maximum (FWHM) to the peaks of the main phase (Fig. 6b–e). Similar small peaks were found in all samples, most notably in the 2.9 < *d* < 3.3 region.

The relative intensities of the extra peaks increase as the Zn content increases, indicating that stronger superstructural ordering is induced by the addition of more Zn. The peaks can be indexed in a primitive trigonal unit cell with *a* ≈ 14.2 Å and *c* ≈ 17.4 Å, which is related to the unit cell parameters of the rhombohedral phase mentioned earlier (Fig. 6c). The presence of (100), (301), (310), and (313) peaks indicates that the superstructure does not order rhombohedrally, but instead orders in a primitive trigonal symmetry of the *P*3 family, *i.e. P*3, *P*3, *P*321, *P*312, *P*3*m*1, *P*3*m*1, *P*31*m*, or *P*31*m*. Significant time was spent developing a primitive trigonal model for these systems, but none could be refined lower than *R*_1_ = 0.12, due to the weak intensities of the superstructural reflections. The appearance of superstructural ordering indicates that the Ba–Cu–Zn–P clathrate-I system has a preferential distribution of M and P atoms over specific atomic sites, and it is less favorable for the structure to form with a completely mixed occupancy of Cu, Zn, and P in each framework site. To shed more light on this extra ordering we performed local structure analysis using TEM and PDF analyses.

### Transmission electron microscopy

**Fig. 7:**
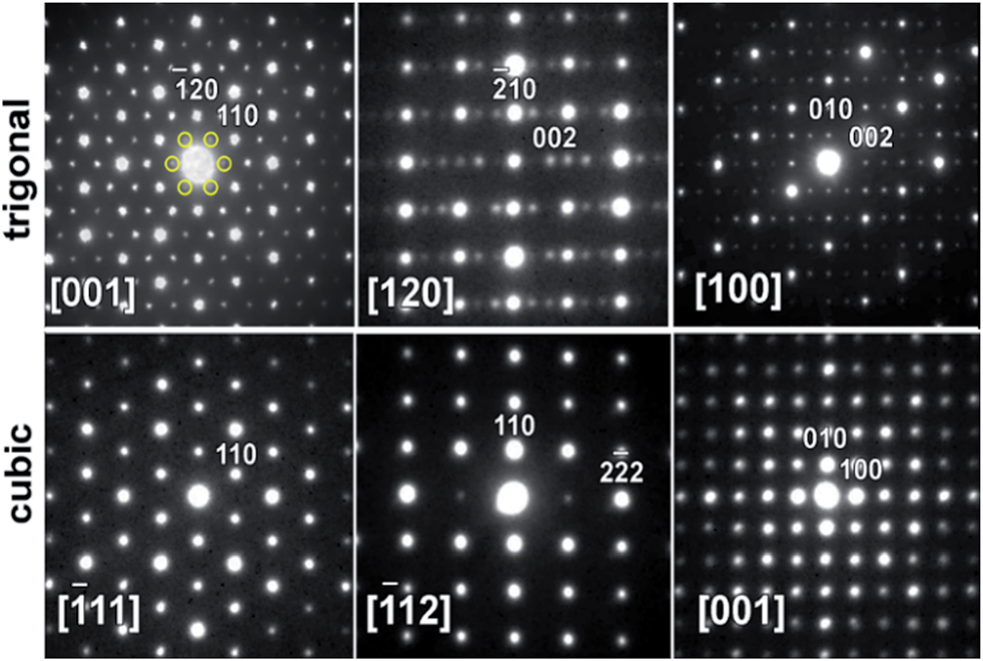
Typical ED patterns are shown along the main zones for (top) trigonal and (bottom) cubic crystallites present in the Ba_8_Cu_11.9_Zn_5.1_P_29_ sample. The six weak (100)-type reflections that violate rhombohedral symmetry are highlighted by yellow circles in the trigonal [001] zone. Note the absence of these and many other reflections in the equivalent direction [–111] in the cubic pattern.

Transmission electron microscopy (TEM) and electron diffraction (ED) were used to study the high Zn-content sample, Ba_8_Cu_11.9_Zn_5.1_P_29_, which had shown clear superstructural peaks in the high-resolution synchrotron X-ray diffraction pattern. Elemental mapping of single crystals shows uniform distributions of the elements over the entire studied areas (Fig. S7†). Based on the results of TEM and ED analyses, the sample appears to be inhomogeneous from a structural point of view with two types of crystallites present in approximately equal amounts. Crystallites of the first type were clearly cubic (*Pm*3*n*) while crystallites of the second type clearly exhibited trigonal symmetry (Fig. 7). The cubic crystals’ diffraction patterns can be completely indexed in the *Pm*3*n* space group determined by XRD. However, the trigonal crystals’ patterns cannot be completely indexed in the rhombohedral cell, as visible from the comparison of the identical directions in clathrate-I, cubic [–111] and trigonal [001] (Fig. 7 left).

**Fig. 8:**
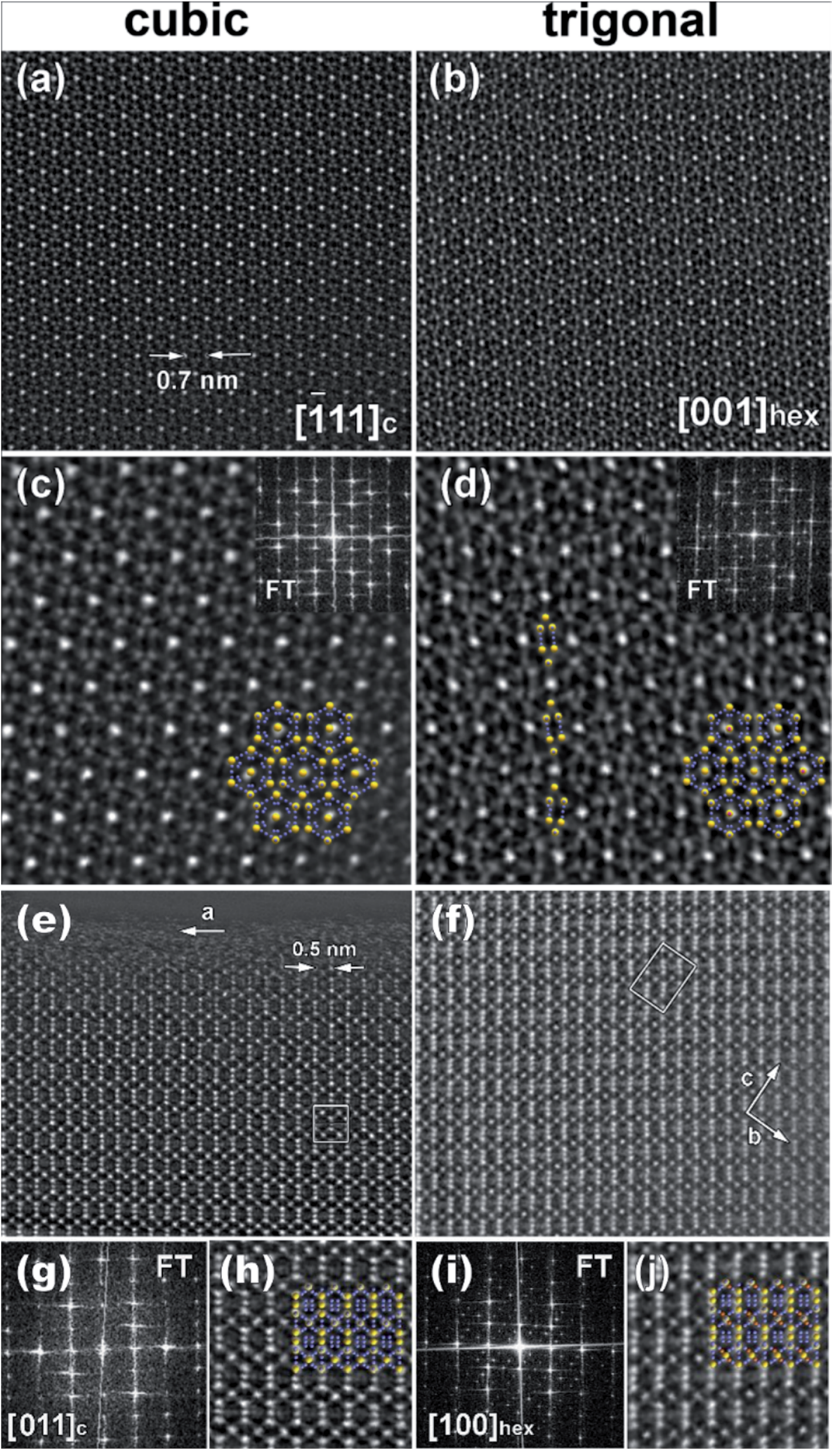
HAADF-STEM images of the (a and c) [–111] cubic and (b and d) corresponding [001] trigonal crystallites. The insets in (c and d) show the FT patterns and the structural fragment overlaps (Ba – yellow, Cu/P – blue). Note the distortion of the Cu–P chain highlighted in (d). (e–j) represent HAADF-STEM images of the [011] cubic (e, g and h) and the corresponding [100] trigonal (f, i and j) crystallites. FT patterns and enlarged images with structural fragment overlays are shown in (g and i) and (h and j), respectively.

Weak reflections, including (100), were reproducibly found in the ED patterns of the trigonal crystallites. These extra reflections may be due to double diffraction, but the presence of extra peaks in the synchrotron X-ray diffraction patterns suggests that additional ordering is present. Based on these results, the sample that looked to be single phase *via* in-house powder X-ray diffraction appears to instead be a mixture of crystallites that are either highly ordered or only partially-ordered. Contrary to what was observed in the clathrate-I Ba_8_Au_16_P_30_,^26^ no intergrowths or macroscopic defects were seen in crystals of Ba_8_M_16+*y*_P_30–*y*_. Fig. 8 and S4† show HAADF-STEM and ABF-STEM images of the Ba_8_Cu_13.1_Zn_3.3_P_29.6_ structure viewed along two main zone axes of the cubic and trigonal structures. The obtained Fourier transformed (FT) diffraction patterns are in good agreement with the observed ED patterns, and they confirm the difference between the two structures, showing superstructure spots. No structural defects, such as antiphase boundaries or stacking faults, were observed for either type of crystallite. The small difference in the atomic arrangements of the trigonal structure from the cubic structure is visible upon a comparative analysis of the Cu–P fragments in the [–111]_cub_ and [001]_hex_ images (Fig. 8c and d). The indicated Cu–P chains are distorted and tilted left and right in the trigonal structure while they are linear in the cubic structure. In the case of the [001]_cub_ and the corresponding [100]_hex_ images (Fig. 8e–j), differences between the two structures occur in the intensities of the Ba columns resulting in the appearance of a superstructure. TEM studies clearly indicate that additional ordering in the “cubic” structure is occurring.

### Synchrotron X-ray and neutron pair distribution function (PDF) analysis

**Fig. 9:**
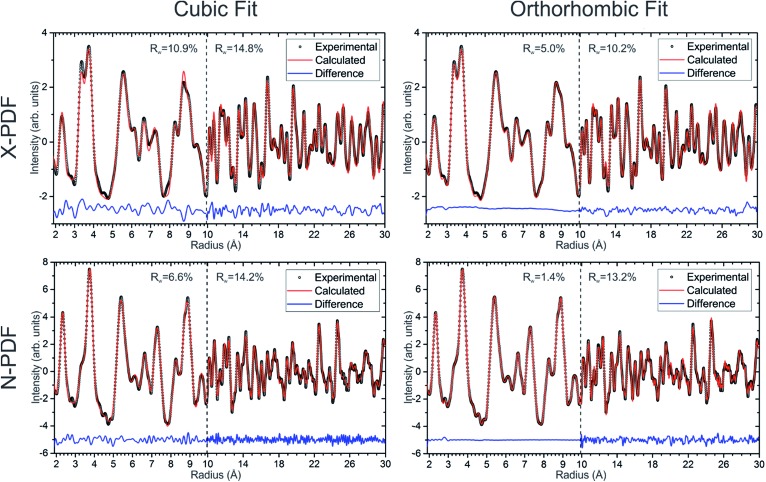
Experimental X-ray (top row) and neutron (bottom row) pair distribution function plots and their fits are shown for cubic (left column) and orthorhombic (right column) models of Ba_8_Cu_13.1_Zn_3.3_P_29.6_. Experimental data are open black circles, calculated fits are red lines, and difference curves are shown as blue lines.

Neutron and X-ray pair distribution function (PDF) analyses were used to probe the local and extended ordering of a single-phase cubic sample of Ba_8_Cu_13.1_Zn_3.3_P_29.6_ (Fig. 9). The 2–4.5 Å range is mainly related to intracage distances. The peaks around 2.2–2.5 Å are attributed to the P–P and M–P distances, while the peaks at 3.3 and 3.7 Å correspond to Ba–M and Ba–P distances, and that of ≈5.4 Å is related to Ba–Ba distances. Additionally, both X-ray and neutron methods show a small peak at ≈2.8 Å whose origin is not inherently obvious in the clathrate-I structure. It can be surmised that this peak is due to either an unexpected (amorphous) impurity phase or may be due to a distortion of the clathrate framework on a local scale. In the orthorhombic fit, the peak at ≈2.8 Å can be fit by a severe distortion of the framework to generate either a short Ba–M distance or a long M–P/M–M distance. This distortion was avoided in the final refinements, keeping a small bump in the difference curves at ≈2.8 Å. The final refinements were conducted such that all bond distances were within a reasonable range; Ba–M/P distances were greater than 3 Å and M/P–M/P distances were less than 2.8 Å, but greater than 2.1 Å.

Short-range (1.9 Å ≤ *r* ≤ 9.9 Å) and long-range (9.9 Å ≤ *r* ≤ 29.9 Å) PDF patterns were fit independently. The short-range data for the Ba_8_Cu_13.1_Zn_3.3_P_29.6_ cannot be satisfactorily fit with the cubic model, indicating that the local structure is more complex than the simple cubic model. Since no structural model is available for the trigonal ordering, we used the fully ordered orthorhombic model, which fits the short-range data much better than the cubic model for both the neutron and X-ray datasets (Fig. 9). In the longer range, 9.9 Å ≤ *r* ≤ 29.9 Å, there is a less obvious improvement of the fit when the data is refined in an orthorhombic model *versus* a cubic model. Similar results were obtained for the cubic samples with different Zn content.

Although refinements using the orthorhombic model may have overparameterization issues, they indicate that the cubic phases do not have completely disordered M/P sites, and that they cannot be well-described by a simple cubic structural model. PDF analyses clearly show that, on the local scale, the cubic structure is additionally ordered and consists of locally-ordered domains which are disordered with respect to one another. According to TEM and XRD those domains have a trigonal structure.

We recently showed that at a higher Cu : Zn ratio of 1 : 1, the clathrate-I structure is unstable, and a new clathrate type, Ba_8_Cu_13_Zn_11_P_28+*δ*_, is formed with a different clathrate-like framework.^29^ Despite having a different framework structure, the Ba_8_Cu_13_Zn_11_P_28+*δ*_ clathrate shares many similarities with the proposed trigonal superstructure for the clathrate-I samples with the highest Zn content, namely, segregation of M and P atoms over different framework positions and the formation of Cu–Zn bonds in the clathrate framework.

## Conclusions

In this work, we scrutinized the Ba_8_M_16+*y*_P_30–*y*_ (M = Cu, Zn) system, which exhibits a high degree of structural complexity. The addition of Zn into the orthorhombic Ba_8_Cu_16_P_30_ clathrate-I framework leads to the structural collapse from an orthorhombic cell with completely segregated M and P sites into a cubic subcell of one quarter of the original size. We show that this collapse is caused not only by a size effect, but also by an electronic effect since the M–P clathrate framework has been shown to accommodate a range of different sized elements without inducing major structural changes, *e.g.* the isostructural Ba_8_Au_16_P_30_.^26^ Both the size and electronegativity of Zn define the preferential occupancies of the Cu sites in the orthorhombic clathrate framework, where Zn is found in the two Cu sites with the shortest Ba–M distances.

The clathrate framework evolves to maintain a better charge balance than that which is obtained from the Ba_8_M_16_P_30_ (M = Cu, Zn) model, allowing excess metal above the semiconducting Ba_8_Cu_14_Zn_2_P_30_ regime. Electron-deficiency is maintained by additional substitutions of Zn for P atoms. Further complexity is observed in the samples with high Zn content that were studied using synchrotron X-ray diffraction, PDF analyses, and TEM. These samples demonstrated the partial segregation of metal and P atoms and the formation of an ordered superstructure, possibly with Cu–Zn bonds, in the clathrate framework.

Overall, our results suggest that complete or partial ordering of small domains is preferred in the Ba_8_M_16+*y*_P_30–*y*_ (M = Cu, Zn) system. Further investigation of the high-temperature thermoelectric properties of these compounds is currently underway.
