# Supplementary material for: Controlling superstructural ordering in the clathrate-I Ba8M16P30 (M = Cu, Zn) through the formation of metal–metal bonds
Source: Chem Sci. 2017 Feb 20;8(5):3650–9. doi: 10.1039/c7sc00354d (PMC5437377; doi:10.1039/c7sc00354d)
Supplement: Supplementary file 1 [file SC-008-C7SC00354D-s001.pdf]

Electronic Supplementary Material (ESI) for Chemical Science.
This journal is © The Royal Society of Chemistry 2017

Supporting Information

Controlling Superstructural Ordering in the Clathrate-I $Ba_8M_{16}P_{30}$ (M= Cu,Zn) through the Formation of Metal-Metal Bonds

J. Dolyniuk,[a] P.S. Whitfield, [b] K. Lee, [a,c] O. I. Lebedev[d] and K. Kovnir[a]

[a] *Department of Chemistry, University of California, Davis, One Shields Avenue, Davis, CA 95616, USA*
[b] *Chemical and Engineering Materials Division, Oak Ridge National Laboratory, Oak Ridge, Tennessee 37830, USA*
[c] *Present Address: Thermal Energy Conversion Technologies Group, Jet Propulsion Laboratory, 4800 Oak Grove Drive, Pasadena, California 91109, USA.*
[d] *Laboratoire CRISMAT, ENSICAEN, CNRS UMR 6508, 6 Boulevard du Maréchal Juin, F-14050 Caen, France*

X-ray and Neutron Powder Diffraction

All samples were characterized by X-ray powder diffraction (XRD) using a Bruker D8 Advance or a Rigaku Miniflex 600 diffractometer employing CuK_{α} radiation. High-resolution synchrotron powder X-ray diffraction data were collected at beamline 11-BM at the Advanced Photon Source (APS), Argonne National Laboratory, using an average wavelength of 0.41 Å. Neutron powder diffraction time-of-flight data were collected at the POWGEN beamline and neutron pair distribution function (PDF) data was collected at the NOMAD beamline at the Spallation Neutron Source (SNS) at Oak Ridge National Laboratory. Additional high-energy total X-ray scattering data (λ = 0.21 Å) were collected at the PDF beamline 11-ID-B at the Advanced Photon Source, Argonne National Laboratory. Neutron powder diffraction data were collected using 1 Å bandwidths centered on 1.333 and 2.664 Å which covered the range 0.3 to 9.2 Å. The datasets were refined simultaneously using TOPAS[1] with structural variables such as lattice parameters, atoms positions, occupancies and displacement parameters constrained across the datasets and instrumental variables such as absorption and background refined independently. Neutron and X-ray PDF were analyzed using PDFGUI.[2,3]

Single Crystal X-ray Diffraction

Single crystal diffraction experiments were carried out at 90 K using a Bruker AXS SMART diffractometer with an APEX-II CCD detector and MoK_{α} radiation. The data sets were recorded as ω-scans at 0.3° stepwidth and integrated with the Bruker SAINT software package.[4] Multiscan absorption corrections were applied due to the irregular shapes of the crystals. The solutions and refinements of the crystal structures were carried out using the SHELX suite of programs.[5] Data for selected samples is shown in Tables S1-S4. Symmetry relationships were evaluated using the Bilbao Crystallographic Server, and these relationships can be seen in Figure S1.[6]

Since Cu and Zn atoms are indistinguishable via single crystal X-ray diffraction, refinements of all compositions were performed with only Cu or P in the framework sites. In the final stages of refinement, the Cu/Zn compositions determined by elemental analysis and/or neutron diffraction were set and not refined further.

All of the measured crystals of $Ba_8Cu_{16-x}Zn_xP_{30}$ selected from samples with nominal compositions $0 \leq x < 1.5$ crystallized in the primitive orthorhombic space group *Pbcn*. For samples with nominal $x \approx 2$ (12.5% Zn/M_{total}), at least two distinct types of crystals were found. Crystals in these samples displayed either the orthorhombic *Pbcn* superstructural unit cell or the primitive cubic *Pm*-3*n* subcell. For higher Zn content samples of $2.5 \leq x \leq 3.3$, single crystals reliably exhibited the cubic *Pm*-3*n* unit cell.

For cubic phases with 12-21% Zn/M_{total}, refinements of Cu/P occupancies resulted in metal-rich, P-deficient compositions, of the form $Ba_8M_{16+y}P_{30-y}$ (M = Cu, Zn). This partial replacement of P atoms with M atoms has strong implications on both the electron count and the transport properties of these materials.

The samples with the highest Zn content from $3.3 < x \leq 5.6$ exhibited more complex structural chemistry. Over this range, strong reflections could be indexed and solved in either the cubic *Pm*-3*n* cell or a rhombohedral *R*-3*c* cell with possible Ba vacancies inside the small dodecahedral cages. However, these models do not fully describe the experimental diffraction data. Synchrotron powder XRD, TEM, and PDF analyses suggested the presence of superstructural ordering in those samples which cannot be fully resolved using conventional single crystal diffraction technique.

To ensure correct symmetry determinations for each system, systematic structural determinations were performed. First, every crystal was solved in the orthorhombic *Pbcn*

cell ($a \approx 14$ Å, $b \approx 10$ Å, $c \approx 28$ Å, $V \approx 4000$ Å^3). This led to one of two scenarios: 1) every M and P site was fully occupied by a single atom type, or 2) every M and P site was jointly occupied by M + P. For samples that fit into 1) the orthorhombic cell was validated. Likewise, for samples following the second scenario, the orthorhombic cell was invalidated and the data was then solved in the cubic *Pm*-3*n* subcell ($a \approx 10$ Å, $V \approx 1000$ Å^3). Finally, for crystals that solved well in the *Pm*-3*n* cell, an *R*-3*c* cell ($a = b \approx 14$ Å, $c \approx 17$ Å, $V \approx 3000$ Å) was tested to check for partial P ordering. Crystals from two samples fit the rhombohedral model with one site fully-occupied by P.

Elemental Analysis

Elemental analyses of at least 10 crystals of each Zn-substituted sample were carried out on a Hitachi S4100T scanning electron microscope (SEM) with energy-dispersive X-ray (EDX) microanalysis (Oxford INCA energy). Elemental analyses were also performed on SPS-pressed pellets, with data collected at a minimum of three locations. Sample analyses confirmed the presence of only Ba, Cu, Zn, and P in all samples.

Transmission Electron Microscopy

Transmission electron microscopy (TEM) samples were ground under ethanol, and the resulting dispersion was transferred to a holey carbon film fixed on a 3 mm copper grid. High-resolution TEM (HRTEM) and electron diffraction (ED) studies were performed using a Tecnai G2 30 UT (LaB_6) microscope operated at 300 kV with 0.17 nm point resolution and equipped with an EDAX EDX detector. High angle annular dark field (HAADF)–scanning TEM (STEM) and annular bright field scanning TEM (ABF-STEM) studies were performed using an JEM ARM200F cold FEG double aberration corrected electron microscope operated at 200 kV and equipped with a large solid-angle CENTURIO EDX detector and Quantum EELS spectrometer.

Density of States Calculations

Density of states calculations were performed with CASTEP using Materials Studio at Oak Ridge National Laboratory.[7] GGA-PBE calculations were performed for the selected phases using ultrasoft potentials on a *k*-point grid of 2×2×1 after ultra-fine geometry optimization of the phases.[8] These calculation results were verified with TB-LMTO-ASA calculations (Figure S2). The band structure and density of states (DOS) for $Ba_8Cu_{16}P_{30}$ (Figure S2) were calculated using the tight-binding, linear muffin-tin orbital, atomic sphere approximation (TB–LMTO–ASA) program.[9] The Barth–Hedin exchange potential was employed for the LDA calculations.[10] The radial scalar-relativistic Dirac equation was solved to obtain the partial waves. A basis set containing Ba(6*s*,5*p*,4*d*), Cu(4*s*,4*p*,3*d*), and P(3*s*,3*p*) orbitals was employed for the self-consistent calculation, with downfolded Ba(6*p*) and P(3*d*) functions. The density of states (DOS) and band structure were calculated after convergence of the total energy on a *k*-mesh with 4×4×2 points, with 18 irreducible *k*-points due to the large size of the orthorhombic unit cell of the compound.

Sample Densification and Physical Properties

Powdered single phase samples of Zn-substituted $Ba_8Cu_{16}P_{30}$ were compacted using spark plasma sintering. 5 mm diameter graphite pressure dies with WC plungers were used as inner dies inside a 20 mm diameter outer die. Samples were compacted at an applied temperature of 923 K (with overheat up to 943 K) and an overall pressure of 204 MPa. An initial 1 kN of force (51 MPa) was applied prior to temperature ramping. Next, samples

were ramped to 923 K over 10 minutes. At 923 K, the force was increased to 4 kN (204 MPa). The maximum temperature and pressure were held for 10 minutes, then the pressure was quickly returned to ambient. Cooled, pressed samples were polished on all sides to remove graphite residue. The resulting geometrical densities of the pellets were greater than 90% of their theoretical X-ray densities. Seebeck coefficients were studied via a commercial Physical Properties Measurement System (Quantum Design PPMS) using the Thermal Transport Option.

1. TOPAS (2015), Bruker, Karlsruhe, Germany
2. T. Proffen and S. J. L. Billinge, *Journal of Applied Crystallography*, 1999, **32**, 572-575.
3. C. L. Farrow, P. Juhas, J. W. Liu, D. Bryndin, E. S. Božin, J. Bloch, P. Th and S. J. L. Billinge, *Journal of Physics: Condensed Matter*, 2007, **19**, 335219
4. *SMART and SAINT*. Bruker AXS Inc.: Madison, WI, USA, 2007.
5. G. Sheldrick, *Acta Crystallographica Section A*, 2008, **64**, 112-122
6. M.I. Aroyo, J.M. Perez-Mato, D. Orobengoa, E. Tasci, G. de la Flor, A. Kirov, *Bulgarian Chemical Communications,* 2011, **43**, 183-197.; b) M.I. Aroyo, J.M. Perez-Mato, C. Capillas, E. Kroumova, S. Ivantchev, G. Madariaga, A. Kirov, H. Wondratschek, *Zeitschrift für Kristallographie,* 2006, **221**, 15-27.; c) M.I. Aroyo, A. Kirov, C. Capillas, J.M. Perez-Mato, H. Wondratschek, *Acta Crystallographica Section A*, 2006**, 62,** 115-128.
7. S.J. Clark, M.D. Segall, C.J. Pickard, P.J. Hasnip, M.J. Probert, K. Refson, M.C. Payne, *Zeitschrift fuer Kristallographie* 2005, **220**, 567-570.
8. J.P Perdew, K. Burke, M. Ernzerhof, *Physical Review Letters,* 1997, **78**, 1396
9. Jepsen, O.; Burkhardt, A.; Andersen, O.K., The Program TBLMTO-ASA, Version 4.7; Max-Planck-Institut für Festkörperforschung: Stuttgart, Germany, 1999.
10. U. V. Barth and L. Hedin, *Journal of Physics C: Solid State Physics*, 1972, **5**, 1629

Table S1. Selected single crystal X-ray diffraction data for orthorhombic crystals of Zn-substituted $Ba_8Cu_{16}P_{30}$.

Formula	$Ba_8Cu_{16}P_{30}$	$Ba_8Cu_{15.1}Zn_{0.9}P_{30}$	$Ba_8Cu_{14.4}Zn_{1.6}P_{30}$
CSD-number	432452	432453	432455
Space Group	*Pbcn* (No. 60)	*Pbcn* (No. 60)	*Pbcn* (No. 60)
Temp (K)	90(2)	90(2)	90(2)
λ [Å]	0.71073	0.71073	0.71073
a [Å]	14.098(1)	14.102(1)	14.1102(7)
b [Å]	10.0785(8)	10.0880(9)	10.0967(5)
c [Å]	27.952(2)	28.023(2)	28.064(1)
V [Å^3]	3971.5(5)	3986.5(6)	3998.2(3)
Volume per formula unit	992.9(1)	996.6(2)	999.6(1)
Z	4	4	4
ρ [g cm^{-3}]	5.092	5.075	5.063
μ [mm^{-1}]	17.362	17.358	17.355
data/param.	4934/247	4938/247	4954/247
R_1	0.020	0.018	0.018
wR_2	0.037	0.036	0.037
Goodness-of-fit	1.06	1.14	1.12
Diff. peak and hole, e/Å^3	0.80 & -0.64	0.79 & -0.58	0.85 & -0.72

Table S2. Selected single crystal X-ray diffraction data for cubic crystals of Zn-substituted $Ba_8Cu_{16}P_{30}$.

Formula	$Ba_8Cu_{13.4}Zn_2P_{30.6}$	$Ba_8Cu_{13.6}Zn_{2.1}P_{30.3}$	$Ba_8Cu_{13.1}Zn_{3.3}P_{29.6}$	$Ba_8Cu_{13.4}Zn_{3.4}P_{29.2}$	$Ba_8Cu_{12.8}Zn_{3.5}P_{29.7}$
CSD-number	432454	432457	432456	432458	432457
Space Group	Pm-3n (No. 223)	*Pm*-3*n* (No. 223)	*Pm*-3*n* (No. 223)	*Pm*-3*n* (No. 223)	*Pm*-3*n* (No. 223)
Temp (K)	90(2)	90(2)	90(2)	90(2)	90(2)
λ [Å]	0.71073	0.71073	0.71073	0.71073	0.71073
a [Å]	10.004(2)	10.008(2)	10.0242(8)	10.028(1)	10.029(2)
V [Å^3]	1001.4(6)	1002.3(5)	1007.3(2)	1008.5(4)	1008.7(5)
Volume per formula unit	1001.4(6)	1002.3(5)	1007.3(2)	1008.5(4)	1008.7(5)
Z	1	1	1	1	1
ρ [g cm^{-3}]	5.022	5.034	5.05	5.063	5.033
μ [mm^{-1}]	17.065	17.199	17.526	17.676	17.419
data/param.	248/9	207/9	248/9	250/9	250/9
R_1	0.028	0.031	0.014	0.019	0.027
wR_2	0.054	0.057	0.031	0.048	0.045
Goodness-of-fit	1.486	1.49	1.46	1.23	1.28
Diff. peak and hole, e/Å^3	0.79 & -0.71	0.88 & -0.89	0.51 & -0.94	0.73 & -0.81	0.74 & -1.00

Table S3. Selected single crystal X-ray diffraction models for other crystals of Zn-substituted $Ba_8Cu_{16}P_{30}$.*

Formula	$Ba_{7.80(6)}Cu_{11}Zn_{5.2}P_{29.8}$	$Ba_{7.80(8)}Cu_{10.4}Zn_{5.6}P_{30}$	$Ba_8Cu_{11.9}Zn_{5.1}P_{29}$	$Ba_8Cu_{11.7}Zn_{5.3}P_{28.9}$
Space Group	*Pm*-3*n* (No. 223)	*Pm*-3*n* (No. 223)	*R*-3*c* (No. 167)	*R*-3*c* (No. 167)
Temp (K)	90(2)	90(2)	90(2)	90(2)
λ [Å]	0.71073	0.71073	0.71073	0.71073
a [Å]	10.0617(8)	10.0684(8)	14.213(1)	14.2150(7)
c [Å]	-	-	17.415(2)	17.4315(9)
V [Å^3]	1018.6(2)	1020.7(2)	3046.6(6)	3050.4(3)
Volume per formula unit	1018.6(2)	1020.7(2)	1015.5(2)	1016.8(1)
Z	1	1	3	3
ρ /g cm^{-3}]	4.963	4.479	5.025	5.026
μ [mm^{-1}]	16.923	14.854	17.591	17.645
data/param.	298/13	252/13	795/37	776/38
R_1	0.022	0.024	0.020	0.019
wR_2	0.047	0.058	0.036	0.042
Goodness-of-fit	1.34	1.50	0.77	1.06
Diff. peak and hole, e/Å^3	0.59 and -0.67	0.87 and -1.02	0.80 and -0.80	0.93 and -0.98

* The data given here are approximate models which do not fit the data completely. The clathrate phases with high Zn content were shown to have additional superstructural ordering aside from *Pm*-3*n* and *R*-3*c* models. In the Ba-deficient cubic phases, 4 out of 5 sites are mixed occupancy or partially occupied, leading to difficulties in accurate structural determinations due to scaling issues.

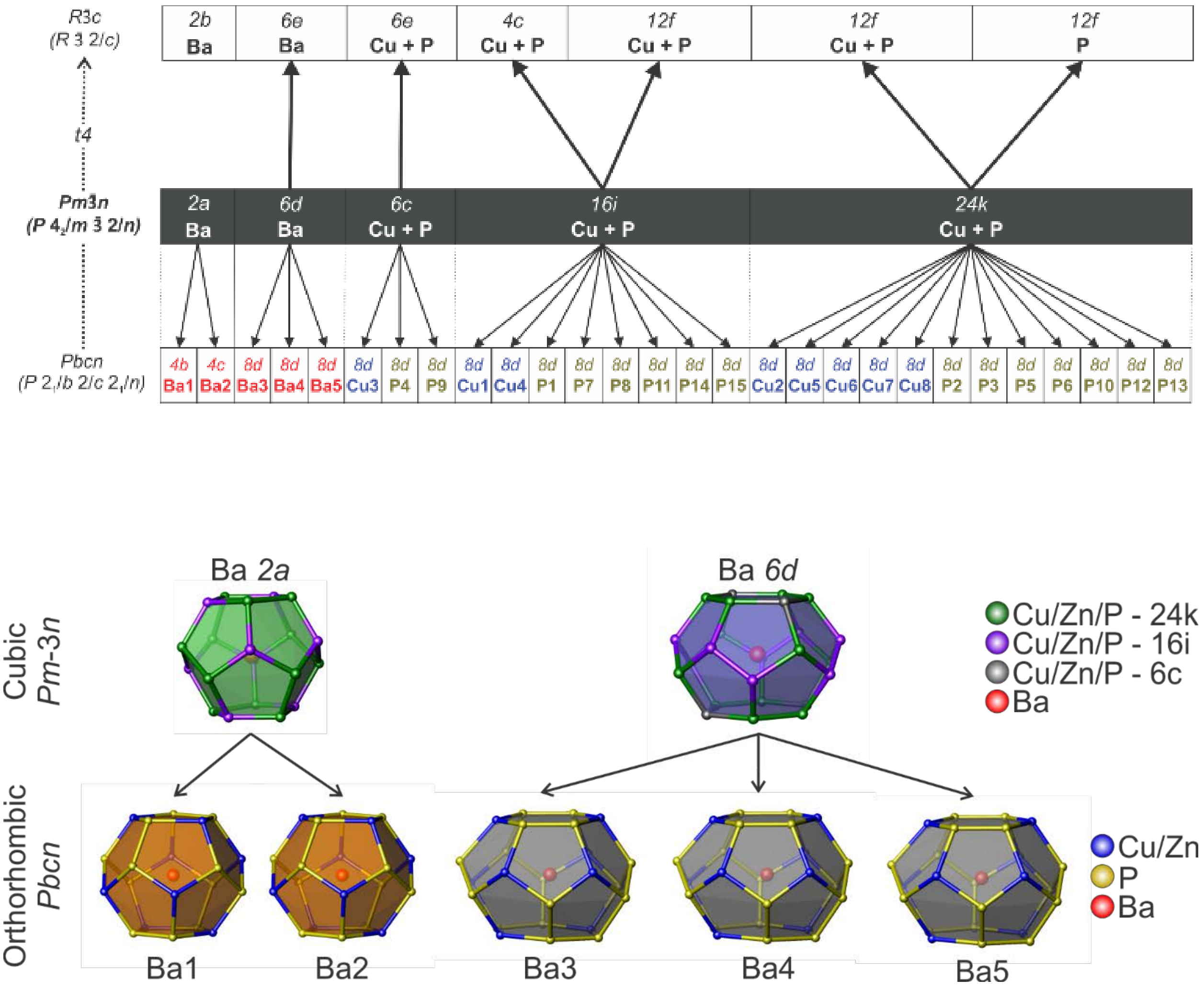

Figure S1. Top: The relationship between space group conformations, i.e., splitting of the atomic positions is shown from cubic to rhombohedral (top) and from cubic to orthorhombic (bottom). A full splitting diagram for the transition from cubic *Pm*-3*n* to *Pbcn* can be found in reference 12 of the main text. Bottom: color coded relationships between atomic positions and polyhedra types in cubic and orthorhombic structures. Color of atoms is identical to Figure 4 in the main text.

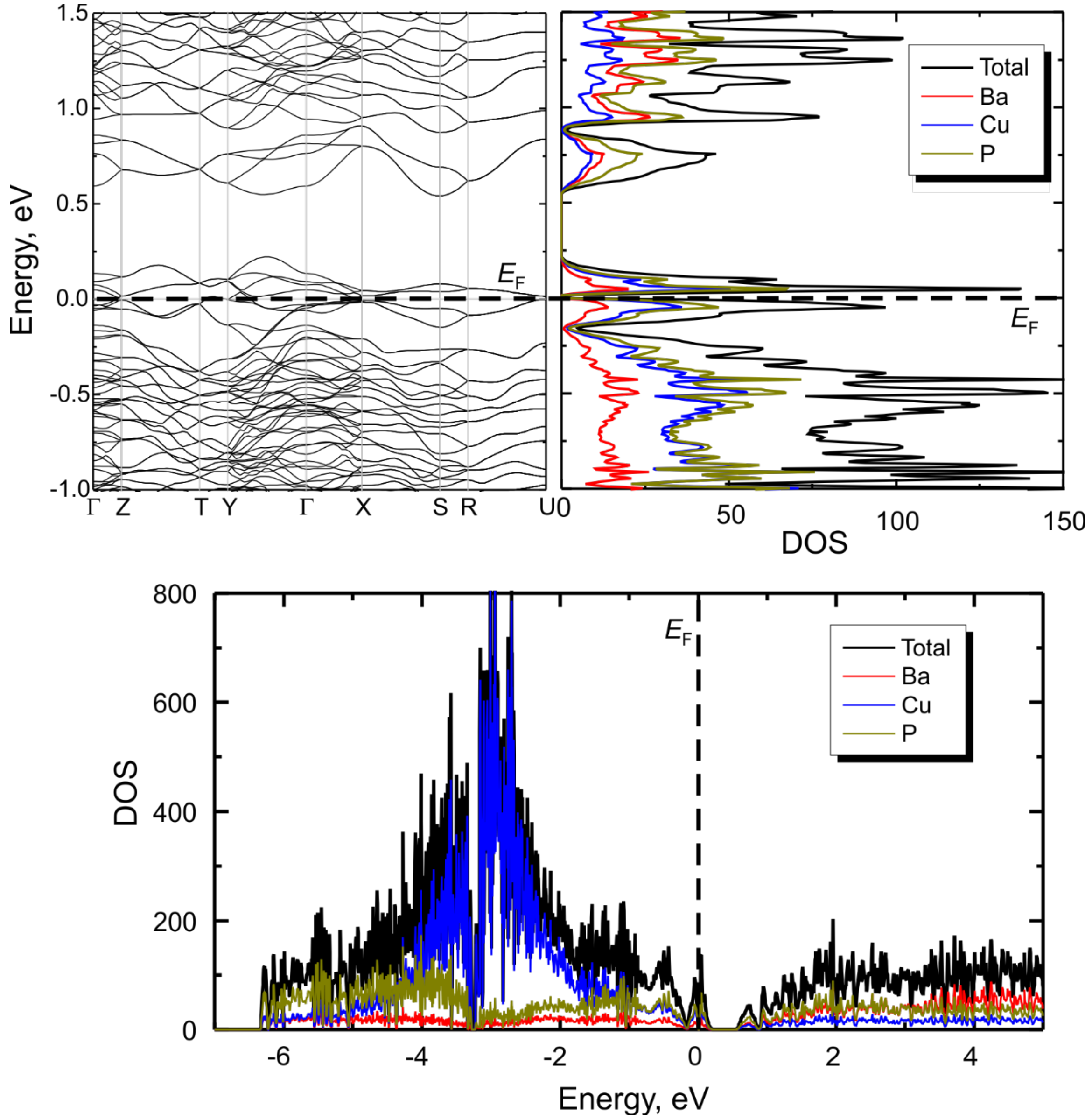

Figure S2. Band structure (top left) and density of states plots (top right and bottom) calculated for $Ba_8Cu_{16}P_{30}$ by the TB-LMTO-ASA method. The integrated area from 0 eV to the band gap is equivalent to approximately 2 electrons per formula unit.

Table S4. Selected cage parameters and bond distances for orthorhombic and cubic crystals.

		$Ba_8Cu_{16}P_{30}$	$Ba_8Cu_{15.1}Zn_{0.9}P_{30}$	$Ba_8Cu_{13.5}Zn_{1.9}P_{30.6}$	$Ba_8Cu_{11.4}Zn_{6.5}P_{28.4}$	$Ba_8Cu_{11.7}Zn_{5.3}P_{28.9}$
	**Type**	**Orthorhombic**		**Cubic**		**Rhombohedral**
**Cage A**	**Coordination**	20	20	20	20	20
	**Average Bond Length (Å)**	3.26	3.27	3.27	3.29	3.29
	**Polyhedral Volume (Å^3)**	98.83	99.19	99.82	101.57	101.32
**Cage B**	**Coordination**	24	24	24	24	24
	**Average Bond Length (Å)**	3.58	3.59	3.59	3.6	3.61
	**Polyhedral Volume (Å^3)**	136.97	137.56	137.80	140.44	139.91
**Distances**	**Shortest Ba-Ba (Å)**	4.8403(7)	4.8339(7)	5.0020(8)	5.0342(4)	5.0279(8)
	**Shortest Ba-P (Å)**	3.1578(9)	3.1618(8)	3.214(2)	3.235(2)	3.2280(9)
	**Shortest Ba-M (Å)**	3.1689(5)	3.1693(4)			
	**Shortest M-M (Å)**	3.6822(8)	3.6795(6)			
	**Shortest P-M (Å)**	2.273(1)	2.2632(8)			
	**Longest P-M (Å)**	2.458(1)	2.4568(8)			
	**Shortest P-P (Å) ***	2.158(1)	2.159(1)	2.235(4)	2.250(4)	2.216(4)
	**Longest P-P (Å) ***	2.477(2)	2.475(2)	2.401(3)	2.433(3)	2.433(1)
	*** P-P represents framework-framework distance for cubic and rhombohedral samples.**					

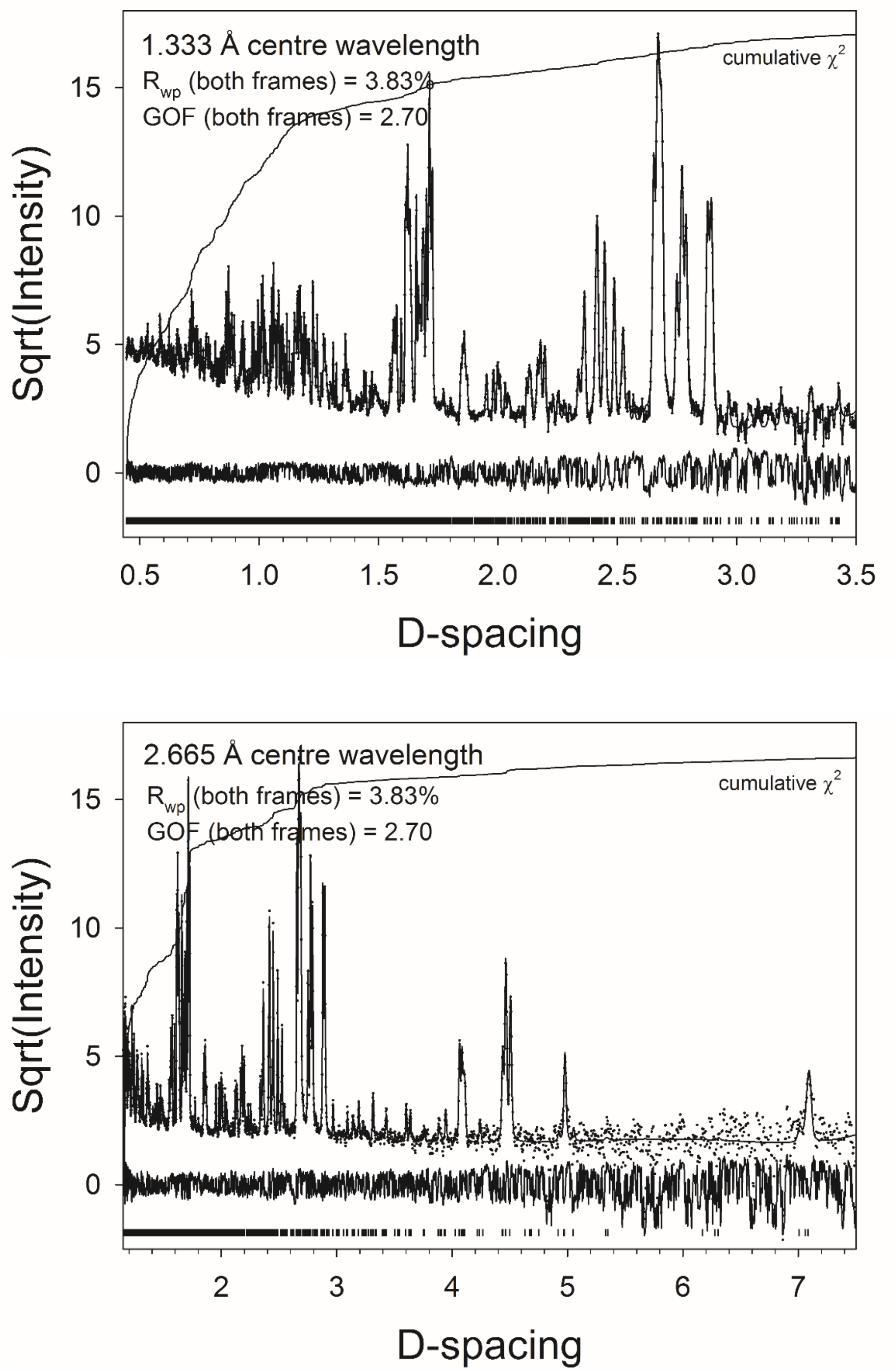

Fig S3. Joint neutron and synchrotron X-ray Rietveld refinement for orthorhombic $Ba_8Cu_{16}P_{30}$.

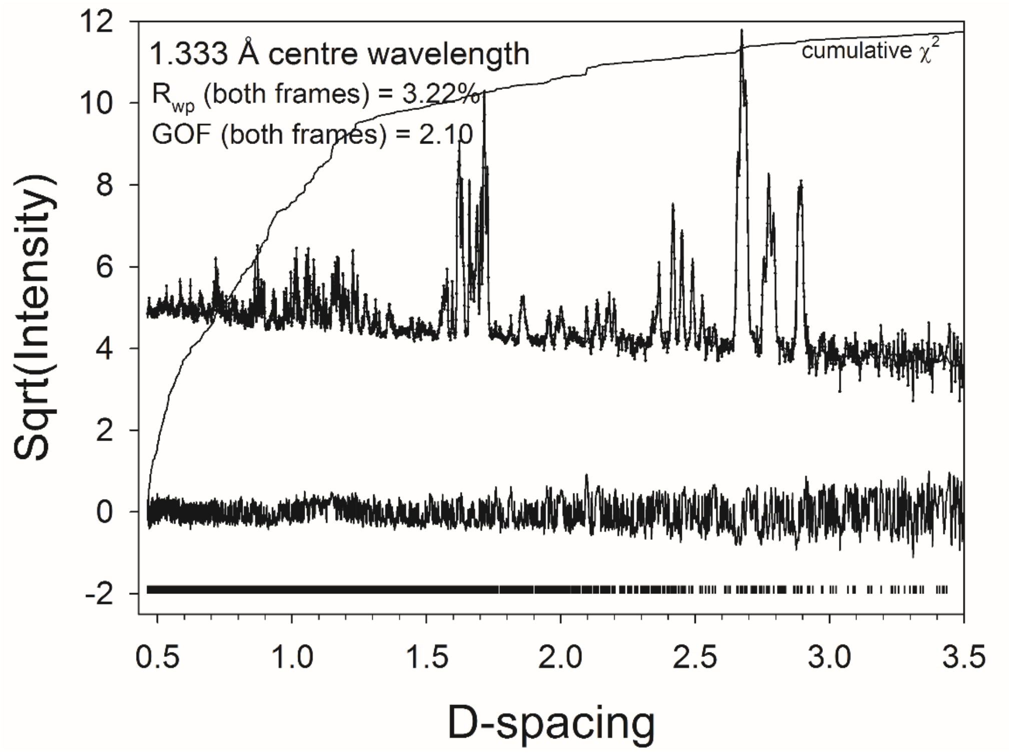

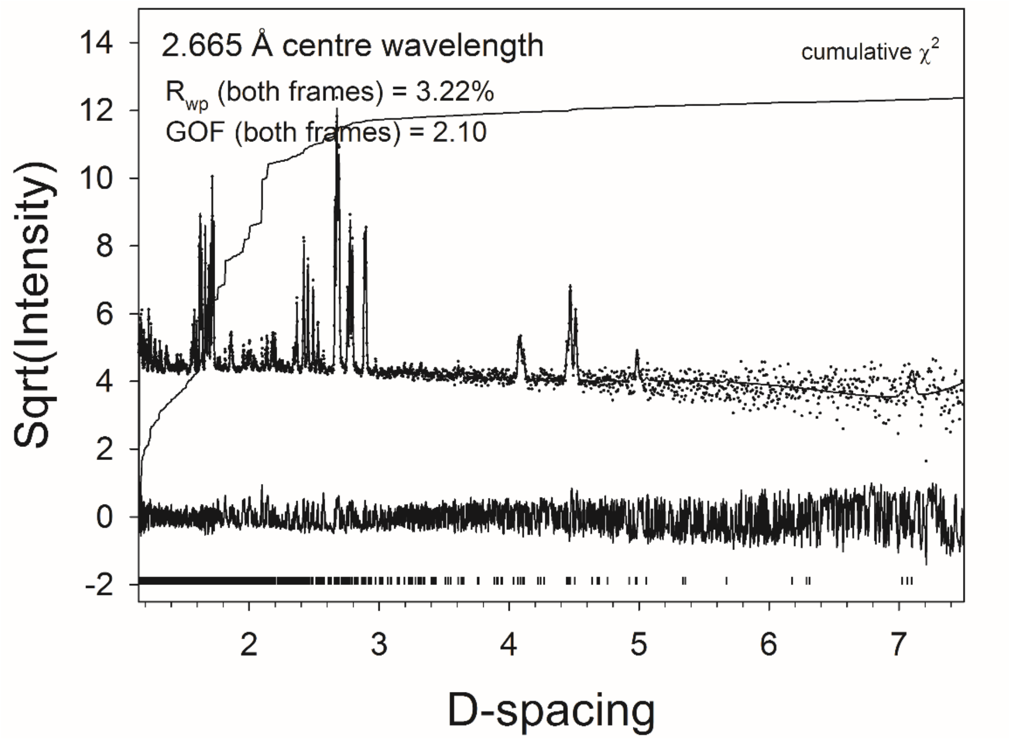

Fig S4. Joint neutron and synchrotron X-ray Rietveld refinement for orthorhombic $Ba_8Cu_{15.1}Zn_{0.9}P_{30}$.

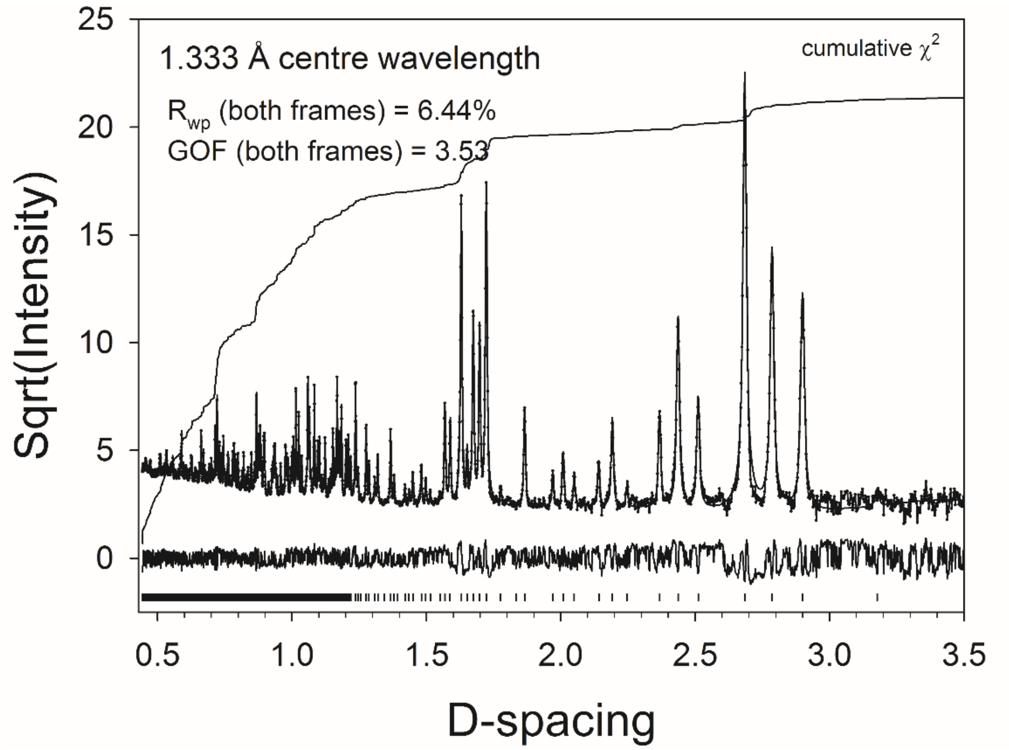

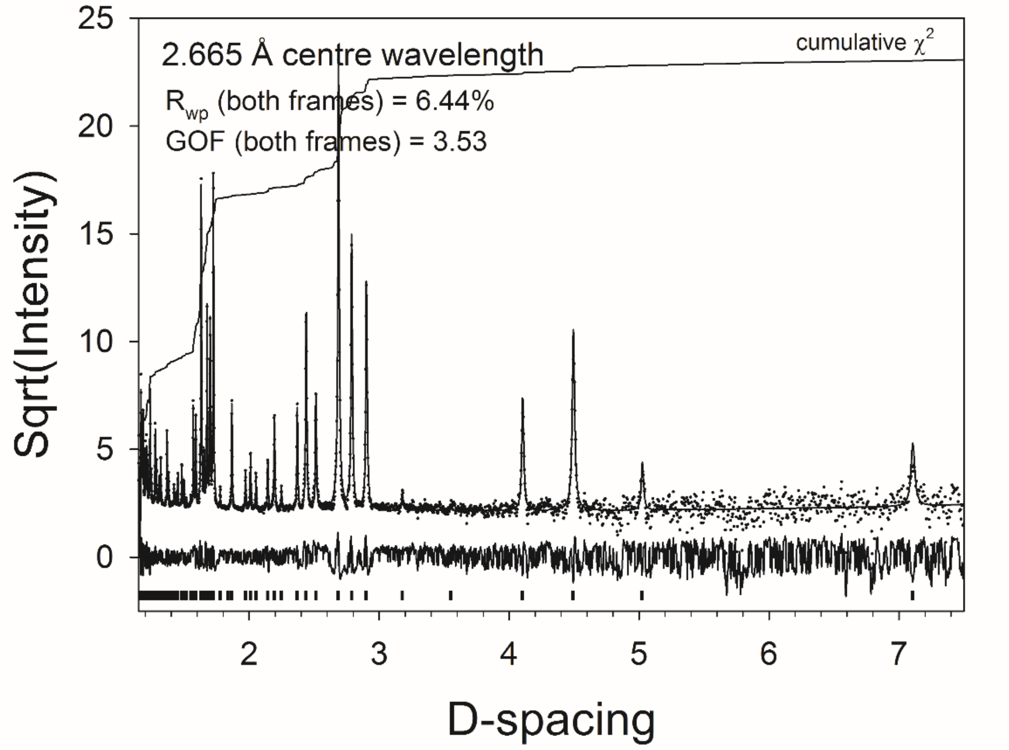

Fig S5. Joint neutron and synchrotron X-ray Rietveld refinement for cubic $Ba_8Cu_{13.1}Zn_{3.3}P_{29.6}$.

Table S5. Refined unit cell parameters for joint refinements of neutron and synchrotron data (left column) and neutron data refined from X-ray coordinates (right column).

Formula	$Ba_8Cu_{15.1}Zn_{0.9(2)}P_{30}$	$Ba_8Cu_{13.1}Zn_{3.3}P_{29.6}$
Space Group	*Pbcn* (No. 60)	*Pm*-3*n* (No. 223)
Temp (K)	300(2)	300(2)
a [Å]	14.13146(7)	10.04608(8)
b [Å]	10.11128(8)	-
c [Å]	28.09101(8)	-
V [Å^3]	4013.84(4)	1013.89(2)
Z	4	1

***Table S6*.** A summary of the joint neutron and synchrotron X-ray powder diffraction Rietveld refinements.

$Ba_8Cu_{15.1}Zn_{0.9(2)}P_{30}$

	Free coordinates		Constrained coordinates	
**Site**	**Cu**	**Zn**	**Cu**	**Zn**
Cu1	0.71(5)	0.29(5)	0.786(13)	0.21(1)
Cu2	1	0	1	0
Cu3	1	0	1	0
Cu4	0.93(7)	0.07(7)	0.80(2)	0.20(2)
Cu5	0.91(3)	0.09(3)	0.96(3)	0.04(3)
Cu6	1	0	1	0
Cu7	1	0	1	0
Cu8	1	0	1	0

$Ba_8Cu_{13.1}Zn_{3.3}P_{29.6}$

**Site**	**Cu**	**Zn**	**P**	**Cu/Zn**
M1 (6c)	0.22(2)	0.10(2)	0.68	2/1
M2 (16i)	0.15(2)	0.07(2)	0.78	2/1
M3 (24k)	0.39(2)	0.07(2)	0.54	6/1

Table S7. Refined coordinates occupancies from joint neutron and synchrotron X-ray data for $Ba_8Cu_{15.1}Zn_{0.9(2)}P_{30}$.*

Site	*x/a*	*y/b*	*z/c*	*Occupancy*	*Multiplicity*	B_{iso}
Ba1	0	0.5	0	1	4	0.226(5)
Ba2	0	-0.008(1)	0.25	1	4	0.180(4)
Ba3	0.1182(4)	0.507(1)	0.3173(2)	1	8	0.493(5)
Ba4	0.1285(3)	0.0133(7)	0.44178(9)	1	8	0.421(5)
Ba5	0.2428(7)	0.250(1)	0.1221(3)	1	8	0.554(6)
Cu1	0.0033(2)	0.3169(5)	0.4079(1)	0.71(5)	8	0.228(5)
Zn1	0.0033(2)	0.3169(5)	0.4079(1)	0.29(5)	8	0.228(5)
Cu2	0.0928(2)	0.007(2)	0.14278(6)	1	8	0.235(5)
Cu3	0.1224(5)	0.4965(7)	0.1896(1)	1	8	0.251(6)
Cu4	0.1886(5)	0.183(2)	0.2519(2)	0.93(7)	8	0.236(6)
Zn4	0.1886(5)	0.183(2)	0.2519(2)	0.07(7)	8	0.236(6)
Cu5	0.2861(3)	0.0083(8)	0.0445(1)	0.91(3)	8	0.227(6)
Zn5	0.2861(3)	0.0083(8)	0.0445(1)	0.09(3)	8	0.227(6)
Cu6	0.3480(4)	0.1225(9)	0.4231(4)	1	8	0.254(6)
Cu7	0.347(1)	0.3757(4)	0.3266(1)	1	8	0.282(5)
Cu8	0.4457(4)	0.3128(2)	0.0326(1)	1	8	0.252(5)
P1	0.0005(7)	0.193(2)	0.3413(6)	1	8	0.26(1)
P2	0.0554(3)	0.310(2)	0.2240(2)	1	8	0.28(1)
P3	0.0660(3)	0.1995(3)	0.0285(5)	1	8	0.24(1)
P4	0.1213(5)	0.013(1)	0.0607(2)	1	8	0.24(1)
P5	0.153(1)	0.119(1)	0.3306(3)	1	8	0.24(1)
P6	0.163(1)	0.383(2)	0.4237(2)	1	8	0.23(1)
P7	0.1819(7)	0.303(2)	0.4993(3)	1	8	0.27(1)
P8	0.1896(6)	0.311(1)	0.0001(1)	1	8	0.28(1)
P9	0.257(2)	0.247(1)	0.3782(3)	1	8	0.26(1)
P10	0.282(1)	0.5006(6)	0.20079(5)	1	8	0.28(1)
P11	0.3176(6)	0.3233(2)	0.2455(4)	1	8	0.27(1)
P12	0.4032(5)	-0.011(2)	0.1067(3)	1	8	0.25(1)
P13	0.4395(7)	0.196(2)	0.2169(3)	1	8	0.26(1)
P14	0.498(1)	0.3154(8)	0.1571(6)	1	8	0.28(1)
P15	0.4991(6)	0.1709(7)	0.0968(3)	1	8	0.25(1)

* Zn was refined in every Cu site and zero occupancy was found for Cu2, Cu3, Cu6, Cu7, and Cu8 sites.

Table S8. Refined occupancies from neutron data for $Ba_8Cu_{13.1}Zn_{3.3}P_{29.6}$.

Site	*x/a*	*y/b*	*z/c*	*Occupancy*	*Multiplicity*	B_{iso}
Ba1	0	0	0	1	2	0.45(1)
Ba2	0.25	0.5	0	1	6	1.22(1)
Cu1	0.25	0	0.5	0.22(2)	6	0.52(6)
Zn1	0.25	0	0.5	0.10(2)	6	0.52(6)
P1	0.25	0	0.5	0.68*	6	0.52(6)
Cu2	0.18546(5)	0.18546(5)	0.18546(5)	0.15(2)	16	0.52(6)
Zn2	0.18546(5)	0.18546(5)	0.18546(5)	0.07(2)	16	0.52(6)
P2	0.18546(5)	0.18546(5)	0.18546(5)	0.78*	16	0.52(6)
Cu3	0	0.30837(6)	0.12058(6)	0.39(2)	24	0.52(6)
Zn3	0	0.30837(6)	0.12058(6)	0.07(2)	24	0.52(6)
P3	0	0.30837(6)	0.12058(6)	0.54*	24	0.52(6)

* Phosphorus site occupancies was fixed to the values obtained from single crystal X-ray analysis and not refined.

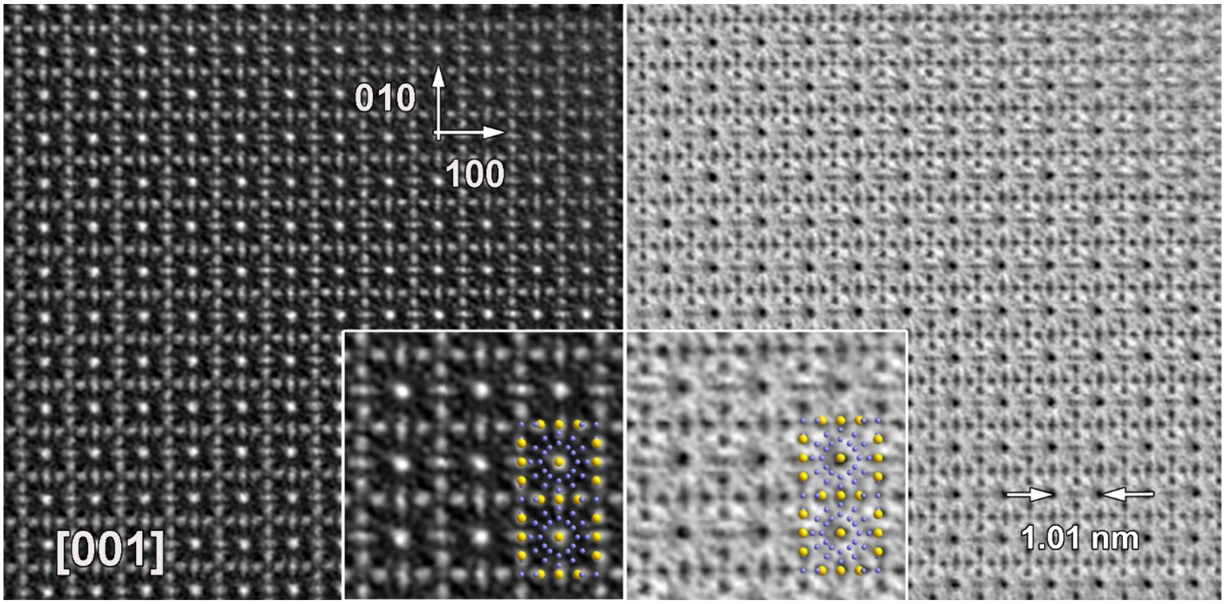

Figure S6. [001] high resolution HAADF-STEM (left) and simultaneously acquired ABF-STEM (right) images of cubic $Ba_8M_{16+y}P_{30-y}$ (M = Cu,Zn) crystal. Enlargements with overlaid structural model are given as inserts (Ba atoms: yellow, Cu/Zn/P: blue).

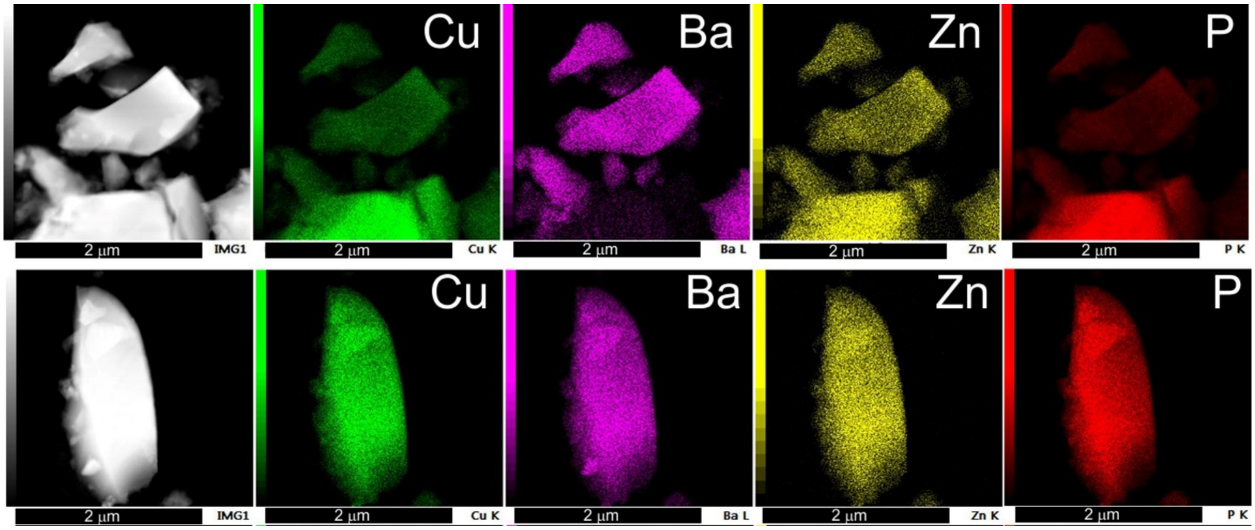

Figure S7. Elemental mapping of the selected crystallites of cubic $Ba_8M_{16+y}P_{30-y}$ (M = Cu,Zn) showing no segregation of the elements.
